# Long non-coding RNA lncMGC mediates the expression of TGF-β-induced genes in renal cells via nucleosome remodelers

**DOI:** 10.3389/fmolb.2023.1204124

**Published:** 2023-05-30

**Authors:** Mitsuo Kato, Zhuo Chen, Sadhan Das, Xiwei Wu, Jinhui Wang, Arthur Li, Wei Chen, Walter Tsark, Ragadeepthi Tunduguru, Linda Lanting, Mei Wang, Roger Moore, Markus Kalkum, Maryam Abdollahi, Rama Natarajan

**Affiliations:** ^1^ Department of Diabetes Complications and Metabolism, Arthur Riggs Diabetes and Metabolism Research Institute, Beckman Research Institute of City of Hope, Duarte, CA, United States; ^2^ Department of Biological Sciences, Indian Institute of Science Education and Research (IISER) Mohali, Mohali, Punjab, India; ^3^ Integrative Genomics Core, Beckman Research Institute of City of Hope, Duarte, CA, United States; ^4^ Transgenic Mouse Facility, Center for Comparative Medicine, Beckman Research Institute of City of Hope, Duarte, CA, United States; ^5^ Department of Immunology and Theranostics, Arthur Riggs Diabetes and Metabolism Research Institute, Beckman Research Institute of City of Hope, Duarte, CA, United States

**Keywords:** long non-coding RNA, lncMGC, diabetic kidney disease, nucleosome remodelers, SMARCA5, epigenetic regulation

## Abstract

**Background:** MicroRNAs (miRNAs) and long non-coding RNAs (lncRNAs) play key roles in diabetic kidney disease (DKD). The miR-379 megacluster of miRNAs and its host transcript lnc-megacluster (lncMGC) are regulated by transforming growth factor-β (TGF-β), increased in the glomeruli of diabetic mice, and promote features of early DKD. However, biochemical functions of lncMGC are unknown. Here, we identified lncMGC-interacting proteins by *in vitro*-transcribed lncMGC RNA pull down followed by mass spectrometry. We also created lncMGC-knockout (KO) mice by CRISPR-Cas9 editing and used primary mouse mesangial cells (MMCs) from the KO mice to examine the effects of lncMGC on the gene expression related to DKD, changes in promoter histone modifications, and chromatin remodeling.

**Methods:** *In vitro*-transcribed lncMGC RNA was mixed with lysates from HK2 cells (human kidney cell line). lncMGC-interacting proteins were identified by mass spectrometry. Candidate proteins were confirmed by RNA immunoprecipitation followed by qPCR. Cas9 and guide RNAs were injected into mouse eggs to create lncMGC-KO mice. Wild-type (WT) and lncMGC-KO MMCs were treated with TGF-β, and RNA expression (by RNA-seq and qPCR) and histone modifications (by chromatin immunoprecipitation) and chromatin remodeling/open chromatin (by Assay for Transposase-Accessible Chromatin using sequencing, ATAC-seq) were examined.

**Results:** Several nucleosome remodeling factors including SMARCA5 and SMARCC2 were identified as lncMGC-interacting proteins by mass spectrometry, and confirmed by RNA immunoprecipitation–qPCR. MMCs from lncMGC-KO mice showed no basal or TGF-β-induced expression of lncMGC. Enrichment of histone H3K27 acetylation and SMARCA5 at the lncMGC promoter was increased in TGF-β-treated WT MMCs but significantly reduced in lncMGC-KO MMCs. ATAC peaks at the lncMGC promoter region and many other DKD-related loci including *Col4a3* and *Col4a4* were significantly lower in lncMGC-KO MMCs compared to WT MMCs in the TGF-β-treated condition. Zinc finger (ZF), ARID, and SMAD motifs were enriched in ATAC peaks. ZF and ARID sites were also found in the lncMGC gene.

**Conclusion:** lncMGC RNA interacts with several nucleosome remodeling factors to promote chromatin relaxation and enhance the expression of lncMGC itself and other genes including pro-fibrotic genes. The lncMGC/nucleosome remodeler complex promotes site-specific chromatin accessibility to enhance DKD-related genes in target kidney cells.

## 1 Introduction

MicroRNAs (miRNAs) and long non-coding RNAs (lncRNAs) play key roles in diabetic kidney disease (DKD) (Kato and Natarajan, 2014; Trionfini et al., 2014; Coellar et al., 2021). The microRNA-379 (miR-379) megacluster of miRNAs and its host transcript, lncRNA and lnc-megacluster (lncMGC), are regulated by transforming growth factor-β1 (TGF-β), increased in the glomeruli of diabetic mice, and promote features of early DKD (Kato et al., 2016). lncMGC was identified in human and mouse glomerular mesangial cells and is upregulated under diabetic conditions such as high glucose (HG) and TGF-β (Kato et al., 2016) treatment and in renal glomeruli of diabetic mice. HG induces TGF-β through the E-box in the promoter of the *TGFb1* gene mediated by transcription factors USF1/2 and ZEB1/2 (Zhu et al., 2005; Kato et al., 2011; Kato et al., 2013). Human lncMGC (hlncMGC) shares exons with other lncRNAs such as *MEG8* and *MEG9* (Charlier et al., 2001; Hatada et al., 2001; Seitz et al., 2004; Kato et al., 2016) and is significantly elevated in plasma samples from individuals with diabetes and diabetic nephropathy compared to healthy controls (Zhang et al., 2020). The hlncMGC was increased by HG treatment in human podocytes (Zhang et al., 2020) and human kidney mesangial cells by TGF-β or HG (Kato et al., 2016), demonstrating that the hlncMGC is upregulated in human kidney diseases. Mouse lncMGC shares exons with other lncRNAs such as *Rian* (RNA imprinted and accumulated in nucleus) and *Mirg* (Seitz et al., 2004). The hlncMGC (*MEG8/MEG9*) and mouse lncMGC (*Rian/Mirg*) are now well-known non-coding RNA accumulated in nucleus (Charlier et al., 2001; Hatada et al., 2001; Seitz et al., 2004; Kato et al., 2016). The abundance of lncMGC RNA is relatively low (three to four copies/cell in the control condition); however, an increase in lncMGC RNA by TGF-β or HG causes a significant increase of ∼40 miRNAs in the cluster and showed a significant impact on the development of DKD (Kato et al., 2016). Although lncMGC is an important regulator of the expression of the miR-379 megacluster as a host transcript, the molecular mechanisms by which it regulates TGF-β actions, genes, and functions related to DKD are not fully understood. Here, we identified nucleosome (chromatin) remodelers, (Clapier et al., 2017) such as SMARCA5 and SMARCC2, as lncMGC-interacting proteins, suggesting lncMGC regulates target genes via epigenetic mechanisms. Notably, our functional studies and integrative Omics profiling of the primary mouse renal mesangial cells from lncMGC-knockout mice revealed novel effects of lncMGC on the expression of TGF-β-regulated genes related to DKD via changes in promoter histone modifications and chromatin remodeling.

## 2 Materials and methods

### 2.1 Identification of lncMGC-interacting proteins

RNA pull down was performed as described (Das et al., 2018) with some modifications. The hlncMGC (GenBank MW802745, MW802746, and MW802747) sense and antisense strands were biotin-labeled after *in vitro* transcription using Biotin RNA labeling Mix (Cat No. 11685597910, Roche) and T3 or T7 RNA Polymerase (Cat No. EP011, Stratagene) from pCR4-TOPO (ThermoFisher Scientific). Biotinylated lncMGC sense and antisense RNAs were treated with RNase-free DNase I and purified on G-50 Sephadex Quick Spin columns (Cat No.11274015001, Roche). Biotinylation efficiency for sense and antisense strands was determined using a Biotin Chromogenic Detection Kit (Cat No. K0661, ThermoFisher Scientific, Carlsbad, CA). 1 μg of biotinylated RNA was denatured by heating to 60°C for 10 min and slowly cooled to 4°C. RNA was mixed with 1 mg of nuclear extract prepared from human kidney cell line HK2 in RNA immunoprecipitation (RIP) buffer (150 mmol/L KCl, 25 mmol/L Tris pH 7.4, 0.5 mmol/L DTT, 0.5% NP40, 100 mmol/L PMSF, and 1x protease inhibitor) and incubated at 4°C for 2 h. Then, 60 μL of washed streptavidin agarose beads (Cat No. SA10004, Invitrogen) was added to each binding reaction and incubated at 4°C for 2 h. The beads were washed five times in spin columns (Cat No. 69725 Pierce, ThermoFisher Scientific). Proteins were eluted using SDS buffer and separated on a 4–15% precast SDS gel (Cat No. 5671084, Criterion, BioRad, Hercules, CA) and stained with SimplyBlue^TM^ SafeStain (Cat No. LC6065, Life Technologies). Bands were excised and subjected to protein identification by the Mass Spectrometry Core at City of Hope National Medical Center. Gel-separated proteins were reduced with DTT (Cat No. R0861, ThermoFisher Scientific, Rockford, IL), alkylated with iodoacetamide (Cat No. A3221-10VL, Sigma, St. Louis, MO), and digested with a mixture of trypsin and LysC (Promega). Peptides were extracted from the gel, evaporated to dryness in a vacuum centrifuge, and resuspended in 0.1% formic acid. The digested peptides were analyzed by LC/MS using an Orbitrap Fusion Mass Spectrometer with an EasyNano1000 nanoflow UHPLC (ThermoFisher Scientific, San Jose, CA). The peptides were loaded onto a 75 µm × 2 cm PepMap trapping column packed with 3 µm C18 silica particles, 100 Å pore size, and then, eluted through a 75 μm × 25 cm PepMap analytical column packed with 2 µm C18 silica particles, 100 Å pore size (both ThermoFisher Scientific), using an 85-min buffer A/B linear gradient from 8% to 25% buffer B (buffer A: 0.1% aqueous formic acid, buffer B: 0.1% formic acid in acetonitrile). The MS spectra of the intact peptides were acquired in the Orbitrap, and the CID MS/MS spectra were acquired in the ion trap. Data were searched using Sequest in Proteome Discoverer 2.1 (ThermoFisher Scientific). The database used was a concatenation of the *Homo sapiens* RefSeq proteome and a database of common laboratory contaminant proteins and was searched separately in the forward (target) and reverse (decoy) direction. The search assumed tryptic specificity with a maximum of two missed cleavages, a precursor ion tolerance of 5 ppm, and a fragment ion tolerance of 0.6 Da. It assumed the quantitative carbamidomethylation of cysteine and potential oxidation of methionine and acetylation of the protein amino terminus. The search results were loaded into Scaffold version 4.8.4 for probability assignment. For mouse lncMGC, all of the procedures used were the same as hlncMGC except that mouse lncMGC (GenBank MW802743 and MW802744) and the mouse kidney cell line TCMK1 were used. The interaction of proteins identified by mass spectrometry was analyzed using STRING DB (https://string-db.org/).

### 2.2 *In vitro* transcription/translation assay

*In vitro* transcription/translation assay was performed to determine the coding potential of lncMGC using the T7 TNT quick-coupled transcription/translation system (Promega, Madison, WI, Cat. No. L1170) and transcend colorimetric non-radioactive translation detection system (Promega, Cat. No. L5070) following the manufacturer’s instructions. In brief, lncMGC lncRNA was subjected to *in vitro* transcription and translation from the full-length pCR4-TOPO construct. Then, 1 µL of the *in vitro* transcription/translation product was added to 15 µL of the SDS loading buffer, followed by protein denaturation at 90°C for 10 min and resolved using 4–15% SDS-PAGE gel. Protein products labeled with the biotinylated transcend tRNA were transferred and detected with streptavidin antibody and Western blue reagent following the manufacturer’s instructions. The T7 control DNA plasmid provided with the kit was used as a positive control.

### 2.3 Generation of lncMGC-KO mice

All animal studies were conducted according to protocols approved by the Institutional Animal Care and Use Committee at the Beckman Research Institute of City of Hope National Medical Center. CRISPR gRNA design software (CRISPR direct) was used to choose the sgRNAs targeting lncMGC, as mentioned previously (Naito et al., 2015; Kato et al., 2021). A mixture of two CRISPR single-guide RNAs (sgRNA) flanking the transcription start site of lncMGC (5′-GAG​UUA​GUG​UGG​CCU​UCA​UC-3′ and5′-GCACGGUGCUGAAAGAGAGG-3′ in equal amounts, 50 ng/μL total) and 50 ng/μL Cas9 enzyme (IDT, Coralville, IA, United States of America) was microinjected into the pro-nuclei and cytoplasm of fertilized C57BL/6J 1-cell embryos. The microinjected embryos were implanted into pseudo-pregnant recipient female mice to produce mutant mice. Mutant candidate mice were screened by performing PCR with DNA extracted from the tails of the surviving mice. Several founders had shorter fragments relative to the WT by fragment analysis (Schuelke, 2000; Kato et al., 2021) and the deletion was confirmed by sequencing. The founders confirmed to have the anticipated deletion crossed with WT C57BL/6 mice and subsequent litters tested for germline transmission of the lncMGC deletion. The heterozygotes were crossed with each other to obtain homozygotes. A significant decrease of lncMGC expression in homozygous lncMGC-KO mice was confirmed using qPCR. Several lines of mutants were obtained.

### 2.4 Isolation of mouse glomeruli and preparation of primary mouse mesangial cells

Glomeruli were isolated from freshly harvested mouse kidneys (Kato et al., 2016; Kato et al., 2021). The renal capsules were removed, and the cortical tissue of each kidney was separated manually. The cortical tissue was gently strained through a stainless sieve with a pore size of 200 μm. The glomeruli were collected and filtered sequentially through sieves with pore sizes of 150 and 75 μm. After several washes with cold PBS, the glomeruli remaining on top of the sieve were collected. Pooled glomeruli were centrifuged, and the pellet was collected for RNA, protein extraction, or for preparing MMC according to our reported methods (Kato et al., 2021). MMCs were maintained in Roswell Park Memorial Institute 1640 Medium supplemented with 10% fetal bovine serum and treated with 10 ng/ml TGF-β (R&D Systems) for 24 h. Passages 5–7 were used for experiments (Kato et al., 2016; Kato et al., 2021).

### 2.5 RNA immunoprecipitation assays

RIP assays were performed as previously reported (Kato et al., 2010; Kato et al., 2021). In brief, MMC from WT and lncMGC-KO mice were plated in four 10-cm Petri dishes (70% confluence), washed with PBS, and cross-linked by exposure to 50 J/m^2^ UV using a UV Stratalinker (Stratagene). The cross-linked cells were collected using 200 μL lysis buffer (provided in the miRNA Isolation kit, WAKO) per dish, gently mixed, incubated on ice for 10  min, sonicated (30 s × 5 with 30 s intervals) using a BIORUPTOR (Diagenode), and stored at −80°C. The lncMGC RNA-protein complex was immunoprecipitated with antibodies, IQGAP (Santa Cruz, sc-374307), NUMA1 (LSBio, LS-B12675), SMARCA5 (Boster Biol. Tech., A02687), DBC1 (Abcam, ab215852), BAT2 (Santa Cruz, sc-373747), MYBBP1A (Novus Biol., NB100-61050), YBX1 (LSBio, LS-B12352), KAP1 (Abcam, ab10484), Nucleolin (Abcam, ab134164), and SMARCC2 (Cell Signaling, #12760) by gently rocking at 4°C and washed with lysis buffer three times. RNAs were extracted using phenol-isoamyl alcohol and chloroform, precipitated with ethanol, washed with 70% ethanol, air dried, and dissolved in 10 μL nuclease-free water. The extracted RNAs were subjected to qPCR. The RIP-qPCR results (normalized enrichment) were calculated by the 2^−ΔΔCT^ method (Livak and Schmittgen, 2001) and normalized to input RNA (purified from 5% of the same cross-linked RNA–protein complex).

### 2.6 Chromatin immunoprecipitation assays

ChIP assays were performed as reported (Kato et al., 2013; Kato et al., 2016). MMCs were left untreated or treated with TGF-β (10 ng/ml) and then cross-linked with formaldehyde. These MMCs were cross-linked with formaldehyde (final concentration of 1% in phosphate-buffered saline for 20 min at room temperature) and then quenched with 125 mM glycine (5 min at room temperature). The cross-linked chromatin was sheared and immunoprecipitated with antibodies against H3K27Ac (Abcam, ab4729) and SMARCA5 (Boster Biol. Tech., A02687). The ChIP-qPCR results (normalized enrichment) were calculated by the 2^−ΔΔCT^ method (Livak and Schmittgen, 2001) and normalized to input DNA (purified from 5% of the same cross-linked chromatin).

### 2.7 RNA sequencing and data analysis

RNA sequencing libraries were prepared with KAPA mRNA HyperPrep Kit (Kapa Biosystems, Cat KR1352) according to the manufacturer’s protocol (Kato et al., 2016; Kato et al., 2021). The libraries were validated with the Agilent Bioanalyzer DNA High-Sensitivity Kit and quantified with Qubit followed by cluster generation and sequencing performed on a HiSeq 2500 platform to generate 51 bp single-end reads at the Integrative Genomics Core of City of Hope National Medical Center. The raw sequences were aligned to the mouse reference genome mm10 using TopHat v2.1.1 (Trapnell et al., 2009) with default parameters, and gene-level expression levels of all the mouse RefSeq genes (downloaded from USCS genome Browser, https://genome.ucsc.edu) were counted using HTseq-count (Anders et al., 2015) v 0.6.0 with settings -s reverse (strand-specific) and -a 10 (filtrating poor alignment reads). The counts were normalized by the trimmed mean of M value (TMM) method (Robinson and Oshlack, 2010), and between-group comparisons on the expression of each expressed gene (RPKM >1 in at least one sample) were performed using the Bioconductor package edgeR v.3.20.9 (Robinson et al., 2010). For each comparison, a different score was assigned to each gene, which is a negative-log-transformed *p*-value followed by negating for the downregulated gene (fold change <1). Pre-ranked GSEA analysis was performed on genes ordered by the resulting score (high to low) using GSEA v. 4.0.3 to identify positive/negative-associated KEGG pathways defined in MSigDB. Bubble plots were generated to reflect normalized enrichment score (NES) and enrichment *p*-values of the significant signatures using R package “ggplot2” (v3.2.0). Upregulated genes (total 479 genes) induced by TGF-ß, namely, TGF-ß upregulated genes, in WT MMCs were identified from the expressed genes (with PRKM >1 in at least three samples) with fold change >2 at FDR <5% when comparing the TGF-ß WT *vs.* SD WT group. Among these genes, those whose expression upregulation by TGF-ß were reversed or attenuated by KO were identified by cluster analysis. IPA was applied to differentially expressed genes (DEGs) for gene ontology and pathway analyses. Motif analysis was performed by geneXplain (https://genexplain.com/).

### 2.8 ATAC sequencing and data analysis

A published Omni-ATAC protocol (Corces et al., 2017) was used for cell lysis, tagmentation, and DNA purification. The Tn5-treated DNA was amplified with 10 cycles of PCR in 50 μL reaction volumes. 1.8X AMPure XP bead purification was used for the PCR product cleanup. The libraries were validated with an Agilent Bioanalyzer DNA High Sensitivity Kit and quantified with qPCR. High-throughput sequencing on Illumina Hisq2500 was performed on the libraries followed by image analysis using real-time analysis (RTA) 2.2.38 software to generate paired-end sequences of 101 bp in-length. Raw reads were subsequently aligned to mouse genome mm10 using Novoalign (V3.02.07), followed by filtering reads located on mitochondria, not properly paired, of pair-end length > 2kb, mapping quality <20, or duplicated reads using Samtools. Genrich v0.5 was further applied to the resulting bam files of the three replicate samples in one group to identify the peak regions (open chromatin) for each group. To identify differentially enriched regions between the two groups, the peak regions of the two groups were combined (union) to generate common peak regions. Reads located in each common region were counted in each sample (three replicates per group) followed by quantile and TMM normalization and compared using edgeR. Differentially enriched regions including hyper-enriched regions and hypo-enriched regions for each comparison were identified as the common peak regions with fold change >1.5 and FDR <5%. To present the enrichment difference at the identified decreased–enriched regions between two groups of samples using heatmap, the coverage of each sample was calculated using all the paired reads using readGAlignmentPairs function provided in the GenomicAlignment R package after removing duplicate reads and scaled to a total of 50 million reads. For each hypo-enriched region between KO *vs.* WT under TGF-ß treatment, the 5 kb flanking region relative to its midpoint was divided into 500 bins (sub-regions), each of 20 bp in length. The average coverage of each bin was then calculated, resulting in a matrix with each row representing one region and each column representing one bin (which has the same location relative to the midpoint of the region). The average coverage of the triplicates in each group was calculated and represented by a heatmap. To get an aggregated profile of all the regions to present for each group, the coverage of each bin across all the regions was averaged and presented as a line plot. *De novo* motif analyses were applied to hypo/hyper-enriched regions to identify transcription binding sites enriched in the identified regions using the peak-motifs function in RSAT Metazoa (http://rsat.sb-roscoff.fr/).

### 2.9 ChIP sequencing and data analysis

ChIP-seq libraries were prepared with a KAPA DNA HyperPrep Kit (Kapa, Cat KK 8700) according to the manufacturer’s protocol. 5–10 ng of immunoprecipitated DNA underwent end-repaired A tailing and adaptor ligation. The final sequencing library was produced by 10 cycles of PCR reactions. The libraries were validated with the Agilent Bioanalyzer DNA High Sensitivity DNA Kit and quantified with Qubit and sequenced on Illumina NovaSeq to generate paired-end 101 bp sequences. Raw reads were subsequently aligned to mouse genome mm10 using BWA and peak calls. A bedgraph file for each sample was generated on the aligned reads after extending to 200 bp on each read and scaled to 50 million total reads. To make the heatmap and aggregate profile of H3K27ac signals at the ATAC hypo-enriched regions in KO-TGF-ß *vs.* WT-TGF-ß, the same approach, as described in ATAC-seq data analysis, was applied to obtain the average coverage of H3K27ac signals across each region (5 kb-flanking region relative to the midpoint) for the duplicate samples in each group and then the aggregated coverage across all the regions for each group.

### 2.10 siRNAs

Oligonucleotides for siRNAs and corresponding control oligos were obtained from Integrated DNA Technologies or ThermoFisher Scientific Inc. (Waltham, MA) and used as described (Kato et al., 2009; Kato et al., 2013). In brief, MMCs (∼10^6^/transfection) were transfected with siRNA oligos using an Amaxa Nucleofector (Lonza, Basel, Switzerland) according to the manufacturer’s protocols. siRNAs (double-stranded oligos) targeting mouse SMARCA5 and non-targeting siRNA controls were obtained from ThermoFisher Scientific. MMCs were trypsinized and resuspended in Basic Nucleofection Solution at 1 × 10^7^/ml. Subsequently, 100 μL of cell suspension (1 × 10^6^cells) was mixed with the siRNA or control oligonucleotides or ON-TARGET plus siRNA or NCs (ThermoFisher Scientific). The transfected cells were collected for RNA extraction. RNA was extracted from the cells, and the expression of coding genes, lncMGC, or miR-379 was examined using primers designed for each target.

### 2.11 Real-time qPCR

RT-qPCR analysis was performed as described (Kato et al., 2016; Kato et al., 2021). RNA was extracted using an RNeasy Mini Kit (Qiagen, Valencia, CA). miR-379 quantification was performed using the qScript miRNA cDNA Synthesis Kit (Quanta Biosciences, Gaithersburg, MD) and amplified using PerfeCTa SYBR Green SuperMix (Quanta Biosciences). For miRNAs, specific mature miRNA sequences were used as forward primers, and the universal primer provided in the kit was used as the reverse primer. U6 was used as an internal control. A GeneAmp RNA PCR Kit (Applied Biosystems, Carlsbad, CA) and POWER SYBR Green Mix (Applied Biosystems) were used for mRNA quantification. mRNA expression was normalized to Cypa as an internal control. Sequences of primers used in this study are shown in Supplementary Table S6.

### 2.12 Statistics and reproducibility

Statistical data analyses were performed using GraphPad Prism software (8.2.1). Normal distribution of each sample group was confirmed using the *χ* ^2^-test or Shapiro–Wilk test before comparison between groups. All data were expressed as the mean ± SEM, and statistical analyses were performed using Student’s *t*-tests (two-sided) to compare the two groups or analysis of variance (ANOVA) followed by *post hoc* Tukey’s test to compare multiple groups. For all experiments, the number of replicates is shown in the figure legends. Asterisks indicate significant difference (*p < 0.05, **p < 0.01, ***p < 0.001, and ****p < 0.0001).

## 3 Results

### 3.1 lncMGC-interacting proteins

**FIGURE 1:**
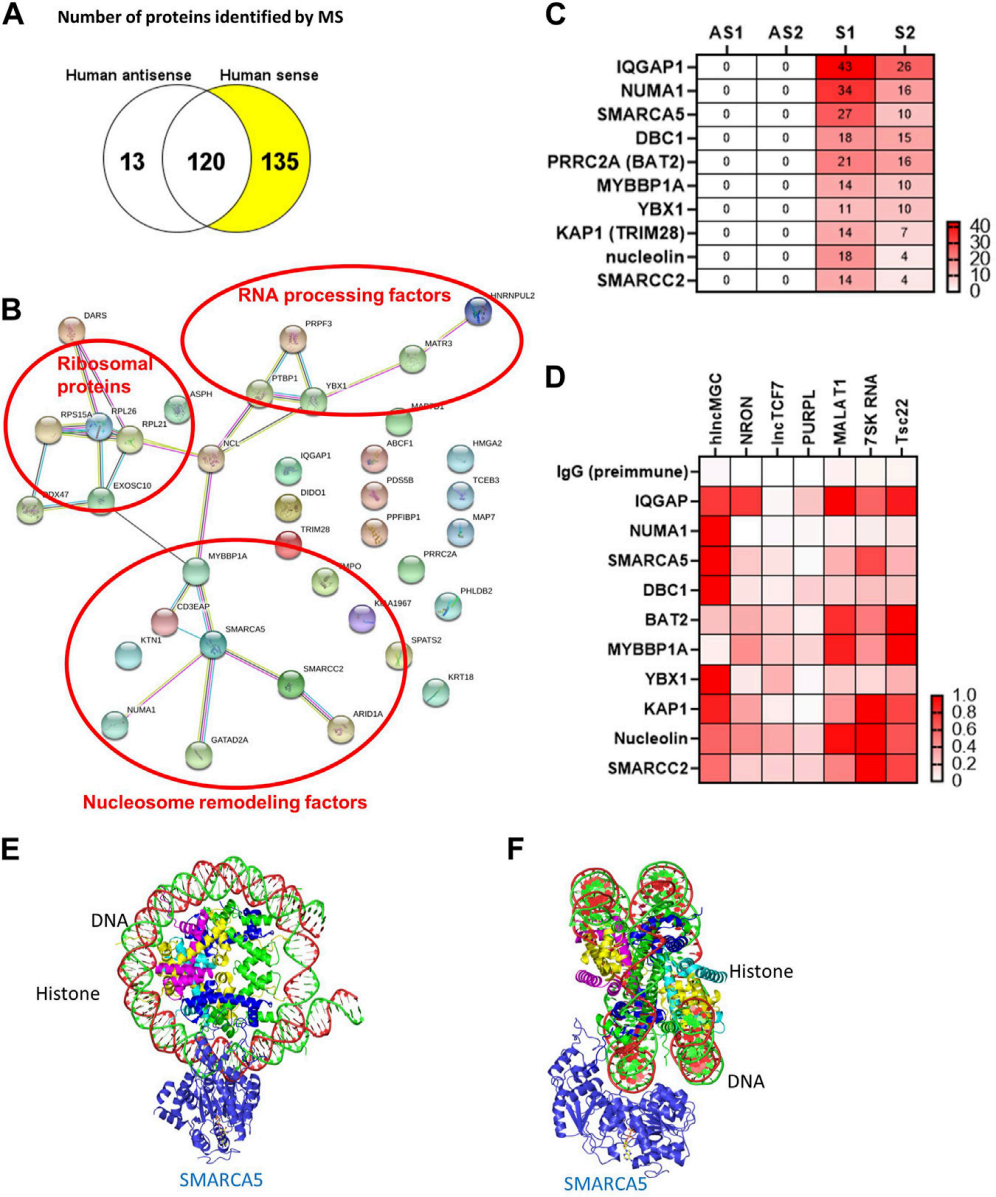
lncMGC-interacting proteins identified by RNA pull down followed by mass spectrometry. **(A)** *In vitro* transcribed RNAs (hlncMGC sense and antisense) were incubated with human HK2 kidney cell lysates and bound proteins isolated and analyzed by mass spectrometry (run in duplicate). 135 proteins were found to interact with sense lncMGC. **(B)** STRING DB (https://string-db.org/) groups the proteins into RNA processing factors (such as PRPF3 and PTBP1), ribosomal proteins (such as RPL26 and RPL21), and nucleosome remodeling factors. **(C)** Heatmap of top 10 candidate proteins based on the number of peptides detected by MS (AS1 and AS2 refer to duplicates from antisense; Supplementary S1 and S2 refer to duplicates from sense). **(D)** RNA immunoprecipitation was used to validate lncMGC binding to the top 10 proteins in HK-2 cells. lncRNA NRON for IQGAP and lncTCF7 for SMARCA5 were used as positive controls (Sharma et al., 2011; Wang et al., 2015). Heatmap of the mean of three independent qPCRs is shown. Normalized affinity was calculated as the ratio of lncRNAs/positive control or as the ratio of lncRNAs/lncRNA with the highest affinity to the target protein. **(E,F)** The structures of SMARCA5 along with histone/DNA are shown in e (from the front) and f (from the side). Protein Data Bank, https://www.ebi.ac.uk/pdbe/entry/pdb/6ne3/analysis#assembly_1.

The genomic structure of hlncMGC (GenBank accession numbers MW802745, MW802746, and MW802747), which covers miR-379 miRNA cluster from miR-379 (most 5′) to miR-656 (most 3’) on human chromosome 14q32.2, is shown in Supplementary Figure S1A (Kato et al., 2016). lncRNA actions are often determined by their protein-interacting partners (Kato and Natarajan, 2019; Statello et al., 2021). Therefore, as a first step, to identify hlncMGC-interacting proteins, hlncMGC (MW802745) was cloned into the pCR4-TOPO expression vector to express lncMGC RNA *in vitro* (Supplementary Figure S1A). No potential lncMGC protein was detected from the vector unlike the positive control luciferase protein, suggesting that hlncMGC RNA is not protein-coding (Supplementary Figure S1B). Very low or no ribosome occupancy (GWIPS-viz, https://gwips.ucc.ie/) (Ingolia et al., 2009) was detected at lncMGC and also other non-coding RNA regions (MEG3), although significant ribosome occupancy was detected at three coding regions (DLK1, PPP2R5C, and DYNC1H1) (Supplementary Figure S1C). Those results again suggest that lncMGC is not a protein-coding RNA, as reported previously (Charlier et al., 2001; Hatada et al., 2001; Seitz et al., 2004; Kato et al., 2016). *In vitro*-transcribed biotinylated hlncMGC RNA was mixed with the human kidney cell (HK2) lysate, and RNA–protein complexes were isolated (Supplementary Figure S2A). Isolated proteins were separated by SDS-PAGE (Supplementary Figure S2B) and analyzed by mass spectrometry (MS). MS data revealed 135 proteins specifically interacted with sense hlncMGC RNA (Figure 1A). The STRING database (https://string-db.org/) suggested that the hlncMGC-interacting proteins identified by MS were grouped into RNA processing factors, ribosomal proteins, and nucleosome remodeling factors (Figure 1B). Interestingly, among the nucleosome remodelers, SMARCA5 was in the center and appeared as a strong candidate hub protein (Figure 1B). The top 10 candidate interacting proteins were displayed in a heatmap (Figure 1C), with the ranking based on the numbers of sense (S) RNA-interacting peptides detected by MS. In HK-2 cells, the interaction of lncMGC and candidate proteins was further validated by RIP and qPCR (Supplementary Figure S2C) and relative affinities depicted as a heatmap (Figure 1D). The relative affinity of hlncMGC for SMARCA5 was higher than that reported for non-coding lncTCF7 (positive control for SMARCA5 interaction) (Wang et al., 2015) (Figure 1D).

We also performed RNA pulldown–MS experiments to identify mouse lncMGC-interacting proteins. The genomic structure of mouse lncMGC (MW802743 and MW802744), which covers the miR-379 miRNA cluster from miR-379 (most 5′) to miR-3072 (most 3’) on mouse chromosome 12qF1, is conserved and displays synteny with the hlncMGC genomic region (Supplementary Figure S3A). Mouse lncMGC (MW802744) was cloned into the pCR4-TOPO expression vector (Supplementary Figure S3B). *In vitro*-transcribed biotinylated lncMGC RNA was mixed with mouse kidney cell (TCMK-1) lysates, and RNA–protein complexes were isolated and analyzed by MS. The STRING DB again suggested groups of RNA processing factors and nucleosome remodeling factors as mouse lncMGC-interacting proteins (Supplementary Figure S4), similar to hlncMGC. SMARCA5 was again selected as a strong candidate because it was in the center of mouse lncMGC-interacting nucleosome remodelers (Supplementary Figure S4). These results suggest that lncMGC regulates the chromatin structure in kidney cells and that the function of lncMGC RNA is conserved from human to mouse. The structure of SMARCA5 along with histone/DNA is depicted in Figure 1E (view from the front) and Figure 1F (view from the side) (Protein Data Bank, https://www.ebi.ac.uk/pdbe/entry/pdb/6ne3/analysis#assembly_1). Through these interactions, SMARCA5 is suggested to unwind nucleosome structures to promote chromatin accessibility and allow gene transcription (Corona and Tamkun, 2004; Erdel and Rippe, 2011). Therefore, hlncMGC–SMARCA5 interactions may regulate chromatin remodeling and enhance the transcription of the lncMGC/miR-379 miRNA cluster as well as other lncMGC targets.

### 3.2 lncMGC-knockout cells to examine molecular actions of lncMGC

To study the molecular actions and function of lncMGC, we used the primary renal mesangial cells isolated from lncMGC-knockout mice, which were generated using CRISPR-Cas9 editing. Two guide RNAs (gRNAs) were designed (Naito et al., 2015; Kato et al., 2021) to delete the upstream region of miR-379 and within lncMGC (Supplementary Figure S5A). Several lines of mutant mice were obtained, and two of them (KO1, 37bp deletion and KO5, 56bp deletion) are schematically shown in Supplementary Figure S5A. Deletion of the indicated genomic region was confirmed by Sanger sequencing (Supplementary Figure S5B). We verified that 56 bp just downstream of INR (initiator, TSS) was missing in lncMGC-KO5 mice. The potential structures of WT and partial lncMGC-KO5 RNAs were predicted by The Vienna RNA Websuite (http://rna.tbi.univie.ac.at/) (Gruber et al., 2008). WT lncMGC RNA, the deleted sequence in the KO5, and partially deleted KO5 lncMGC RNA are shown in Supplementary Figure S5C. Based on our subsequent data, it is likely that the stem-loop structure (partly deleted in the KO5 RNA) may be important for the interaction with nucleosome remodelers such as SMARCA5 and the regulation of chromatin structures. We established primary cultures of MMC from these KO mice and tested the candidate gene expression (Supplementary Figures S5D, S5E). A significantly lower expression of lncMGC, miR-379, and Mirg lncRNA MMC (overlapping with the 3’ part of mouse lncMGC) was confirmed in lncMGC-KO5 compared to the WT MMC. lncMGC was not expressed in lncMGC-KO1 and KO5 MMC. There was a significant increase in lncMGC in the WT MMC after TGF-β (10 ng/ml for 24 h) (Kato et al., 2009; Kato et al., 2011) treatment (Supplementary Figure S5D). Furthermore, the induction of miR-379 and Mirg by TGF-β was also lost or attenuated in lncMGC-KO MMC, whereas both miR-379 and Mirg were increased by TGF-β in the WT MMC (Supplementary Figures S5E, S5F). lncMGC-KO5 MMC, which showed a trend toward lower miR-379 expression than KO1 MMC (Supplementary Figure S5E), was used for further studies.

### 3.3 Transcriptome analysis (RNA-seq) of WT and lncMGC-KO5 MMC reveals TGF-β- and DKD-related functions for lncMGC

**FIGURE 2:**
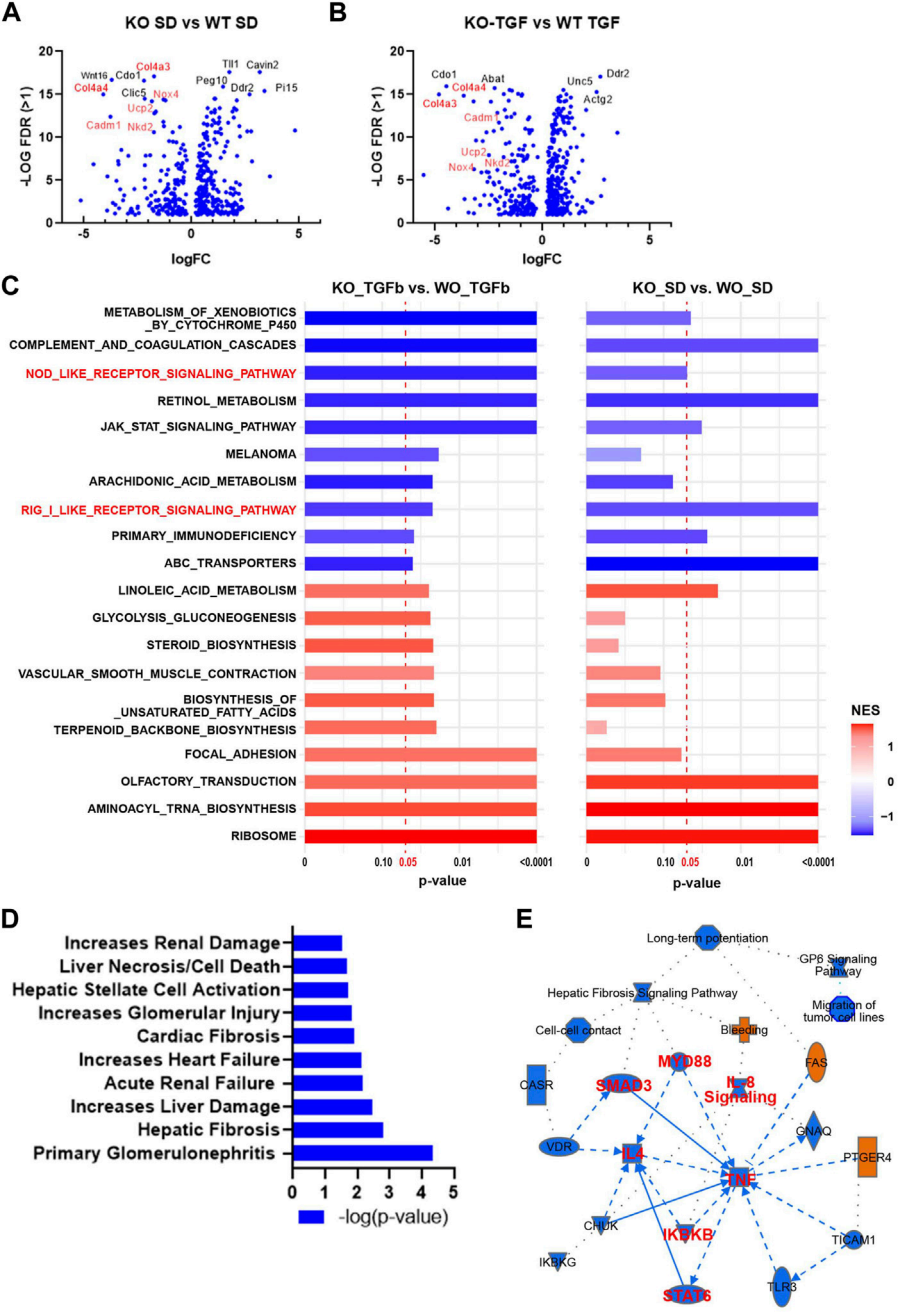
Differentially expressed genes and enriched gene sets and pathways identified by RNA-seq in WT MMC *vs.* lncMGC KO5 MMC. **(A,B)** Volcano plots showing DEGs in KO-SD *vs.* WT-SD (SD refers to serum-depleted control untreated cells) and DEGs in KO-TGF *vs.* WT-TGF. Results show a significant decrease of pro-fibrotic extracellular matrix (ECM) genes such as *Col4a3* and *Col4a4* in KO5 MMC compared to WT MMC at the baseline **(A)** and after TGF-β treatment (10 ng/ml for 24 h) **(B)**. **(C)** GSEA results for DEGs in KO-SD *vs.* WT-SD (left side), and KO-TGF-β-treated *vs.* WT-TGF-β-treated cells (right side). NOD-like receptor gene and RIG-I sets are highlighted (Red). NES, normalized enrichment score. **(D)** Ingenuity Pathway Analysis of DEG analysis (upregulated by TGF-β in WT MMC but decreased in KO5 MMC). IPA of DEG between WT and KO5 MMC (FC > 2, FDR<0.05, rpkm>1) shows enrichment of pathways related to increased renal damage/diseases including glomerular injury, primary glomerulonephritis, acute renal failure, and fibrosis. **(E)** IPA also shows enrichment of genes related to inflammation such as TNF, MYD88, and NF-kB and the TGF-β signal transducer Smad3.

To test the global transcriptomic effects of lncMGC, RNA-seq was performed on MMC isolated and cultured from WT and lncMGC-KO5 mice (Figure 2). The RNA-seq results showed differentially expressed genes with a significant decrease of extracellular matrix (ECM) genes, such as *Col4a3* and *Col4a4*, in KO5 MMC both in control (serum-depleted/SD) and TGF-β-treated cells, confirming that KO5 MMC have attenuated response to TGF-β (Figures 2A, B). Gene set enrichment analyses (GSEA) comparing KO *vs.* WT MMC identified several gene sets related to inflammatory response, such as RIG-I-like receptor, NOD-like receptor, and JAK-STAT signaling, which were attenuated in KO *vs.* WT, basal and TGF-β treated cells (Figure 2C). Furthermore, Ingenuity Pathway Analyses (IPA) of differentially expressed genes (upregulated by TGF-β in WT MMC but downregulated in KO5 MMC), to elucidate DKD related pathways, revealed inclusion of genes associated with kidney damages and fibrosis (Figure 2D) and inflammation (TNF in the center and SMAD3) (Figure 2E).

**FIGURE 3:**
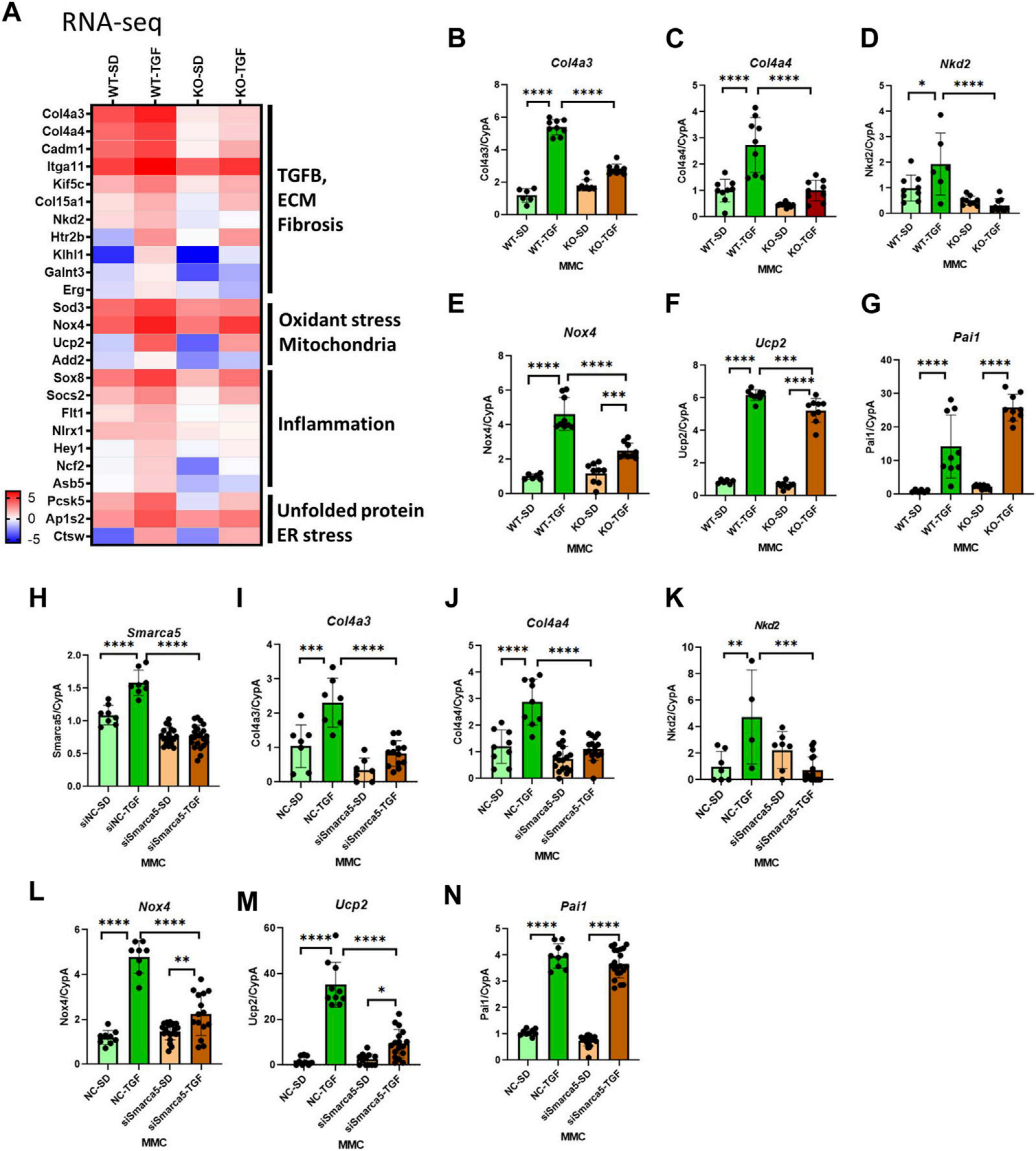
Differentially expressed candidate genes related to DKD from RNA-seq in WT and lncMGC KO5 MMC. **(A)** Heatmap (the mean of Log2RPKM from three independent samples) demonstrated genes associated with DKD among the DEGs. The changes in expression of *Col4a3, Col4a4, Ucp2, Nox4, Nkd2*, and *Pai1* were confirmed by RT-qPCR **(B–G)**. Effects of siSMARCA5 in WT MMC on the expressions of *SMARCA5* **(H)**, *Col4a3* **(I)**, *Col4a4* **(J)**, *Nkd2* **(K)**, *Nox4* **(L)**, *Ucp2* **(M)**, and *Pai1* **(N)**. Data are shown as the mean of three independent experiments calculated from triplicate qPCRs. One-way ANOVA with *post hoc* Tukey’s test for multiple comparisons; ±SEM; *, *p* < 0.05; **, *p* < 0.01; ***, *p* < 0.001; and ****p < 0.0001.

The heatmap shown in Figure 3A emphasized that genes involved in the pathology of DKD, including TGF-β, ECM, fibrosis, oxidative stress, mitochondria, inflammation, and ER stress pathways, are upregulated by TGF-β in WT MMC but attenuated in lncMGC-KO5 MMC, suggesting that lncMGC has widespread effects on gene expression regulated by TGF-β (Figure 3A). We further validated the differential expression of *Col4a3, Col4a4, Ucp2, Nox4,* and *Nkd2,* by RT-qPCR (Figures 3B–G). Significantly lower expression of these genes was detected in KO5 MMC compared to WT MMC in the TGF-β-treated condition by RT-qPCR, although no such decrease was detected in the control condition (SD). *Pai1* expression was not significantly reduced in KO5 MMC (Figure 3G), suggesting not all DKD-related genes may be regulated by lncMGC.

To determine if SMARCA5 is involved in the regulation of key DKD-related genes by lncMGC, we treated WT MMC with SMARCA5 siRNA (siSMARCA5) or a negative control siRNA (siNC) (Figures 3H–N). siSMARCA5 significantly decreased the expression of TGF-β-induced *Col4a3, Col4a4, Ucp2, Nkd2,* and *Nox4* in WT MMC (Figures 3H–M). However, the expression of some genes such as *Pai1* was not affected by siSMARCA5 (Figures 3N) and mirrors data in KO MMC (Figure 3G).

### 3.4 ATAC-seq analysis of WT *vs.* lncMGC-KO5 MMC suggests lncMGC may promote chromatin relaxation at TGF-β- and DKD-related genes

**FIGURE 4:**
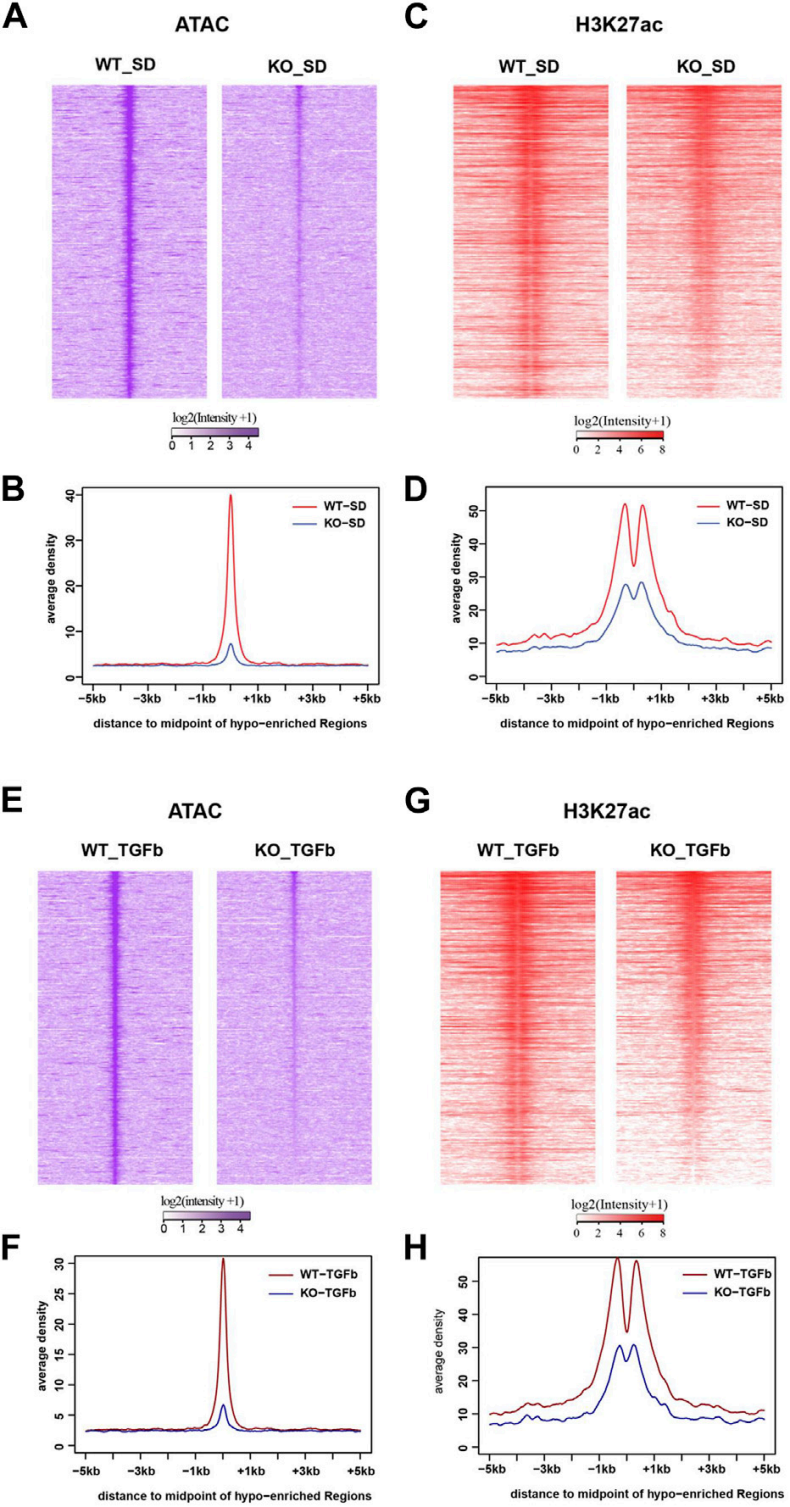
Genome-wide epigenetic regulation in WT and lncMGC KO5 MMC. **(A)**, **(B)** Global ATAC-peaks and H3K27ac peaks at TSS in KO5 MMC compared to WT MMC in the control condition **(A–D)** and TGF-β-treated condition **(E–H)**. Signal intensities, log2 (intensity+1), from upstream (-5 kb) to downstream of TTS (midpoint) of genes identified in this study **(A,C,E, and G)** and average densities are shown **(B,D,F, and H)**. Data are shown as the mean of triplicate ATAC-seq in each condition.

Because of the connection of lncMGC to nucleosome remodeling factors, we next analyzed its impacts on the chromatin structure by genome-wide mapping of chromatin accessible regions (open chromatin) using Assay for Transposase-Accessible Chromatin with high-throughput sequencing (ATAC-seq). The ATAC-seq results identified peaks at 6,919 loci in WT MMC more than KO5 MMC in the control untreated condition. After treatment with TGF-β, 3,862 loci were more accessible in WT *vs.* KO5 MMC, suggesting lncMGC can confer widespread alterations in chromatin and that this process is TGF-β sensitive. There was a significant decrease in global ATAC-peaks (intensities and average densities) from -5 kb to +5 kb at transcription start sites (TSS) in KO5 MMC compared to WT MMC in control (Figures 4A, B) and TGF-β-treated conditions (Figures 4E, F). Since histone H3K27 acetylation (H3K27ac) is a chromatin mark of active enhancers and gene expression (Creyghton et al., 2010; Kundaje et al., 2015) in parallel, we performed chromatin immuprecipitation-Seq (ChIP-seq) with the histone H3K27ac antibody (Figures 4C, D, G, H & Supplementary Figure S6). Similar to ATAC-seq, we observed significant decreases in global H3K27ac-peaks (intensities and average densities) from -5 kb to +5 kb at TSS in KO5 MMC compared to WT MMC in control (Figures 4C, D) and TGF-β-treated conditions (Figures 4G, H).

**FIGURE 5:**
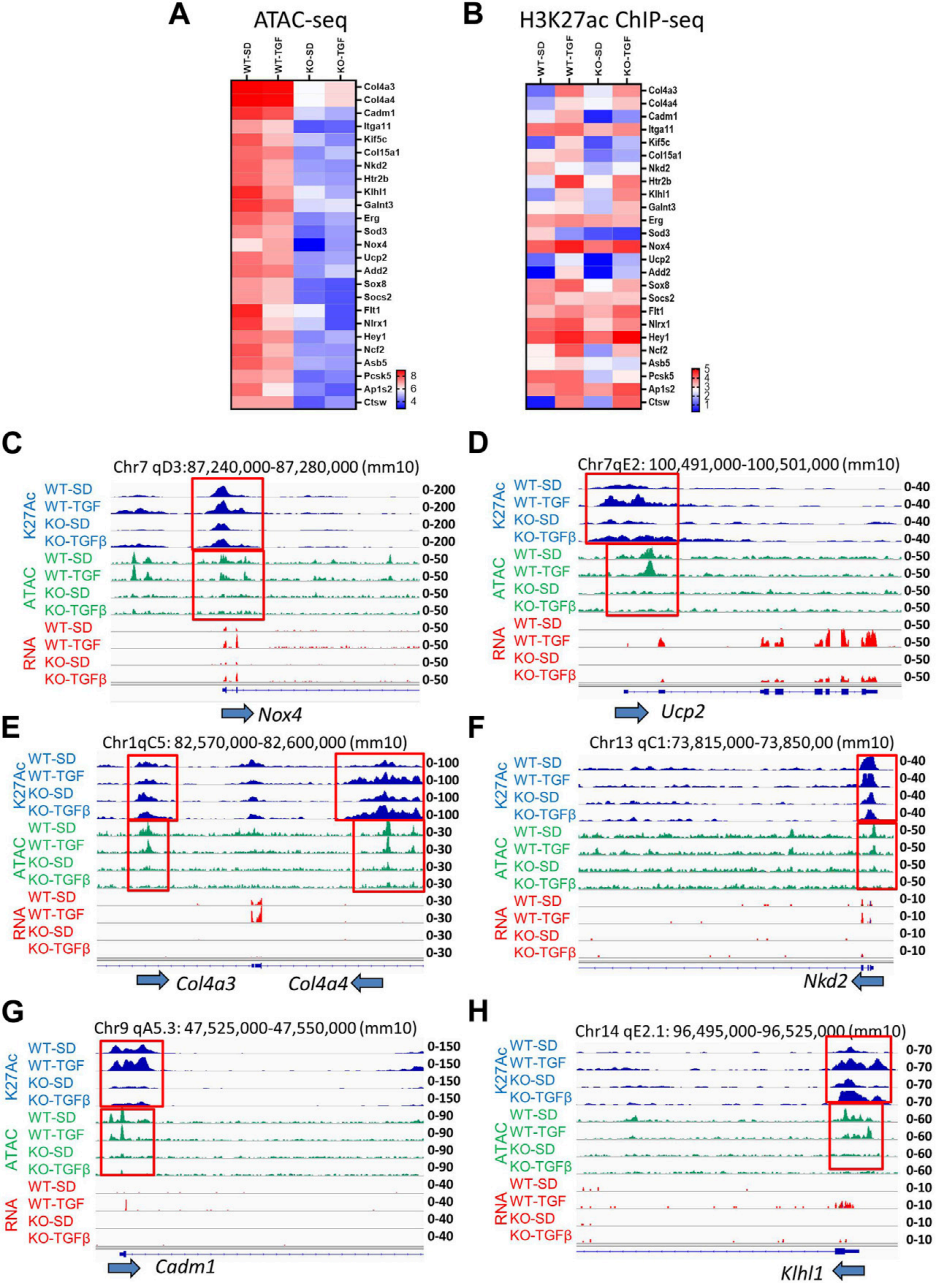
**(A)** Heatmap (the mean of Log2 reads from three independent samples) shows ATAC-seq results at candidate gene loci involved in DKD that were open in WT MMC but closed in lncMGC-KO5 MMC even in the TGF-β-treated condition. **(B)** Histone H3K27ac ChIP-seq in WT and lncMGC-KO5 MMC. Heatmap (the mean of Log2 reads from two independent samples) shows H3K27ac in gene loci involved in DKD that are open in WT MMC but closed in lncMGC-KO5 MMC even in the TGF-β-treated condition. **(C)** Genomic tracks of H3K27ac, ATAC-seq, and RNA-seq at the representative *Nox4* gene locus. The expression of *Nox4* was upregulated by TGF-β in WT MMC but this was significantly lower in KO5 MMC (RNA–seq, red). ATAC-seq tracks showing the promoter regions are open before TGF-β treatment in WT MMC but closed in KO5 MMC even after TGF-β treatment (ATAC-seq, green). H3K27ac was increased by TGF-β treatment in both WT and KO5 MMC (blue). The ATAC-peaks and H3K27ac peaks position relative to the promoter of the *Nox4* gene. **(D–H)** Similar genome tracks shown for other representative genes analyzed include *Ucp2, Col4a3, Col4a4, Nkd2* (Kuppe et al., 2021), *Cadm1* (Hagiyama et al., 2019), and *Klhl1* genes in WT and KO5 MMC (RNA-seq, red) with and without TGF-β (ATAC-seq, green). H3K27ac was increased by TGF-β treatment in both WT and KO5 MMC (blue).

Heatmap ATAC-seq peak analysis indicated that gene loci involved in DKD show higher signals in WT MMC compared to lncMGC-KO5 MMC even after TGF-β treatment. Thus, lncMGC may be involved preferentially in the opening chromatin at the loci of DKD-related genes regulated by TGF-β (Figure 5A). We next checked the signals of ATAC-peaks nearby candidate genes involved in DKD (Figure 3A) and observed a significant decrease of ATAC-seq signals in KO5 MMC (Figure 5A) at genes decreased in KO5 MMC treated with TGF-β (Figure 3A).

We also observed H3K27ac signals at H3K27-enriched regions (H3K27ac-peaks) in the promoter or gene body of the DEGs related to DKD (Figure 3A), which also confirmed the overall correlation of H3K27ac signals with gene expression (Figure 5B) with some exceptions. H3K27ac levels were increased even in KO5 MMC treated with TGF-β. It is likely some TGF-β-regulated genes, such as *Col4a3, Col4a4, Ucp2, Nox4,* and *Nkd2,* were suppressed in KO5 cells (Figure 3A) because their chromatin was closed in the KO5 MMC (Figure 5A) even though H3K27 was acetylated (Figure 5B). Then, we more closely examined the genome tracks of RNA-seq, ATAC-Seq, and H3K27ac ChIP-seq at *Nox4(Oh et al., 2016)*, *Ucp2, Col4a3, Col4a4, Nkd2, Cadm1,* and *Klhl1* representative loci (Figures 5C–H). The expression of *Nox4* and *Ucp2* genes (associated with oxidative stress) was upregulated by TGF-β in WT MMC, but the induction was clearly lower in KO5 MMC (RNA-seq, red). ATAC tracks showed that their promoter regions were open even before TGF-β treatment in WT MMC and closed in KO5 MMC even after TGF-β treatment (ATAC-seq, green). H3K27ac was slightly increased by TGF-β treatment in both WT and KO5 MMC (blue). IGV tracks show the overlap of H3K27ac peaks and ATAC-peaks at the *Nox4* promoter (Figure 5C), confirming ATAC-seq marks at regulatory regions. H3K27ac is an epigenetic mark of active promoters and enhancers (Creyghton et al., 2010; Kundaje et al., 2015). Genes associated with kidney fibrosis, such as *Col4a3, Col4a4, Nkd2* (Kuppe et al., 2021), *Cadm1* (Hagiyama et al., 2019), and *Klhl1*, showed similar trends (Figures 5D–H). Differentially expressed genes (upregulated by TGF-β but downregulated in KO5 MMC), such as Xylt1 (Tziastoudi et al., 2020), Pcsk5 (Petra et al., 2022), and Ap1s2 (Woroniecka et al., 2011; Zhong et al., 2013; Gong et al., 2016), also showed similar patterns in ATAC and H3K27ac (Supplementary Figure S7). *Nlrx1* (related to mitochondrial immunity and inflammation) (Moore et al., 2008; Tattoli et al., 2008; Arnoult et al., 2009; Nagai-Singer et al., 2019) showed significantly lower expression and ATAC-peaks in KO5 MMC (Supplementary Figure S8). This was in line with a more closed chromatin in KO5 MMC and in parallel significantly lower expression of *Nlrx1,* despite relatively high levels of H3K27ac. These results support the notion that the chromatin status dictates the expression of the TGF-β-regulated genes more than H3K27ac. Yet, other genes, such as *Pai1, Ctgf,* and *Il11,* showed high expression in KO5 MMC treated with TGF-β like WT, suggesting not all genes regulated by TGF-β are similarly regulated by lncMGC (Supplementary Figure S9A–S9C).

### 3.5 lncMGC regulates its own promoter

**FIGURE 6:**
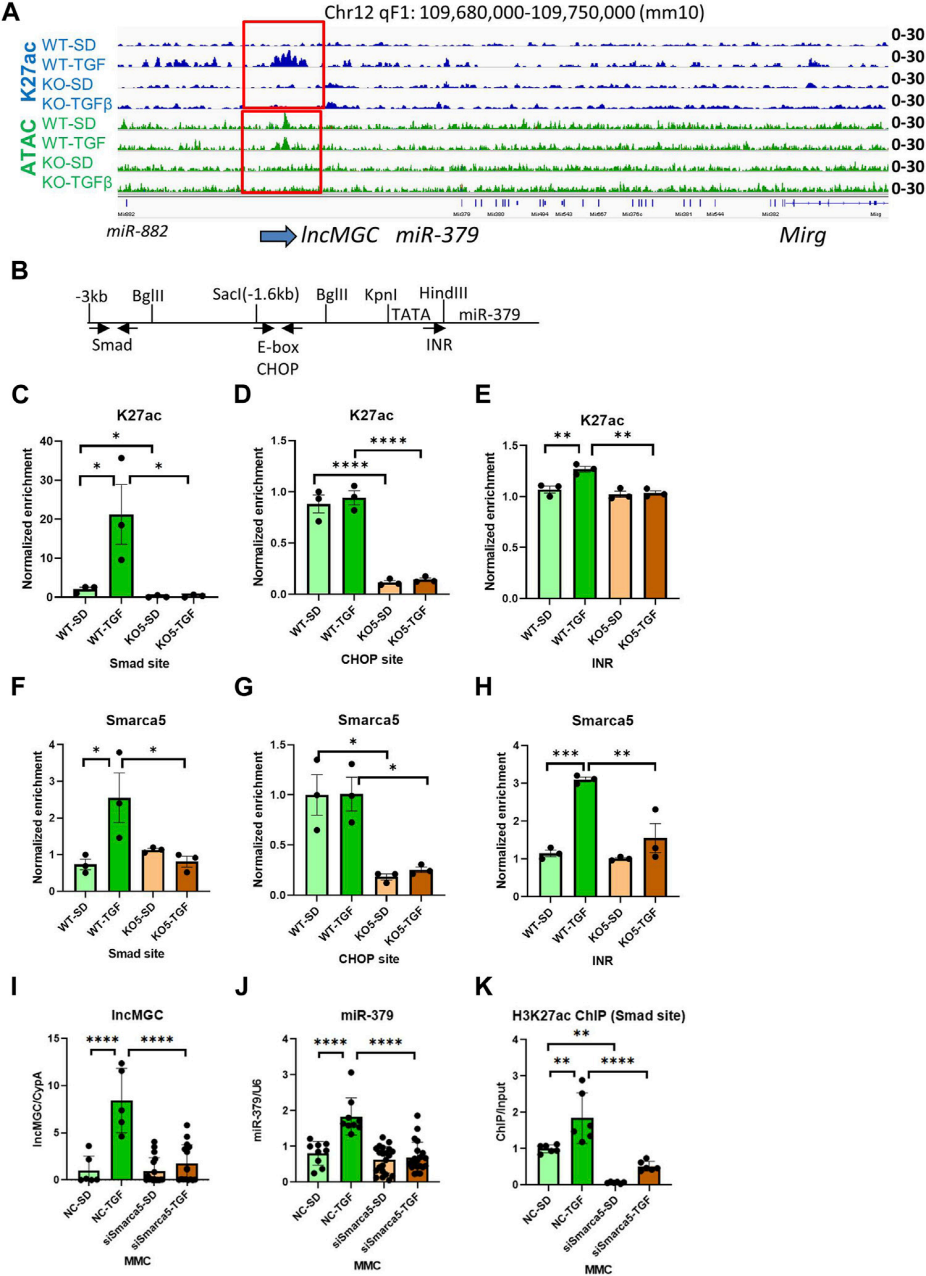
Autoregulation of lncMGC expression. **(A)** H3K27ac at TSS of lncMGC shows clear increase in WT MMC treated with TGF-β but no increase in KO5 MMC under basal or TGF-β-treated conditions. ATAC-seq tracks show a decrease of ATAC-peaks at TSS of lncMGC in KO5 MMC compared to WT MMC in control and TGF-β-treated conditions. **(B)** Schematic of the genomic region of the promoter of mouse lncMGC/miR-379 cluster depicting Smad and CHOP binding sites and INR (TSS). Arrows indicate positions of PCR primers for Smad and CHOP sites. The arrow (INR) shows the position of INR. **(C–H)** H3K27ac and SMARCA5 enrichment (ChIP assays) increased by TGF-β in WT MMC at the lncMGC promoter Smad binding site and initiator (INR) site also known as the transcription start site. However, such an increase was not detected in lncMGC-KO5 MMC, suggesting lncMGC regulation of its own promoter. **(I–K)** Effects of siSMARCA5 on the expression of lncMGC **(I)**, miR-379 **(J)**, and H3K27ac at the Smad site of the lncMGC promoter **(K)**. Data are shown as the mean of three ChIP experiments calculated by triplicate qPCRs each. One-way ANOVA with *post hoc* Tukey’s test for multiple comparisons; ±SEM; *, *p* < 0.05; **, *p* < 0.01; ***, *p* < 0.001; and ****p < 0.0001.

Interestingly, the promoter region of lncMGC showed ATAC-peaks in WT MMC even before treatment of TGF-β, but very low ATAC-peaks in KO5 MMC even after TGF-β treatment (Figure 6A and Supplementary Figure S10). TGF-β increased H3K27ac in WT but not in KO cells (Figure 6A). Therefore, an open chromatin status also appears critical for the expression of lncMGC.

Previously, we identified Smad and CHOP binding sites in the promoter of lncMGC (Kato et al., 2016). Thus, we determined H3K27ac enrichment at these transcription factor binding sites by ChIP-qPCR (Figures 6B–E). Interestingly, H3K27ac levels were increased at the Smad site and initiator (INR, TSS) in WT MMC treated with TGF-β, while no changes were detected in KO5 MMC, again confirming the loss of TGF-β response in the KO5 MMC (Figures 6B–E). TGF-β treatment of WT and KO5 MMC did not alter K27ac enrichment at the CHOP binding site, although levels were significantly lower in untreated KO cells *vs.* WT MMC. These results suggest that lncMGC regulates its promoter.

Given that SMARCA5 is an lncMGC-interacting protein, we performed ChIP assays to determine SMARCA5 enrichment at the Smad and CHOP sites in the lncMGC promoter. SMARCA5 enrichment was increased in WT MMC treated with TGF-β at the Smad site and INR (Figures 6F, H), but not in KO5 MMC even after TGF-β treatment, confirming our finding of increased K27ac at the lncMGC promoter in WT MMC treated with TGF-β but not in KO5 MMC (Figure 6A). At the CHOP site, SMARCA5 binding was decreased in KO5 MMC compared to WT MMC, and TGF-β treatment did not alter SMARCA5 change in either cell type (Figure 6G). Furthermore, the expression of lncMGC and miR-379, as well as the enrichment of H3K27ac at the Smad site in the lncMGC promoter, was significantly increased by TGF-β in siNC-treated control cells but significantly reduced in MMC treated with siSMARCA5 (Figures 6I–K). These results demonstrate that the chromatin around the lncMGC promoter is closed in KO cells and this is accompanied by lower H3K27ac levels and lower expression of lncMGC (and miR-379). These data suggest that lncMGC may regulate its own promoter and other target loci regulated by SMARCA5. Nucleosome remodelers such as SMARCA5 may be recruited to the lncMGC promoter through the interaction with lncMGC RNA to subsequently alter the chromatin structure.

### 3.6 lncMGC regulates neighboring genes

**FIGURE 7:**
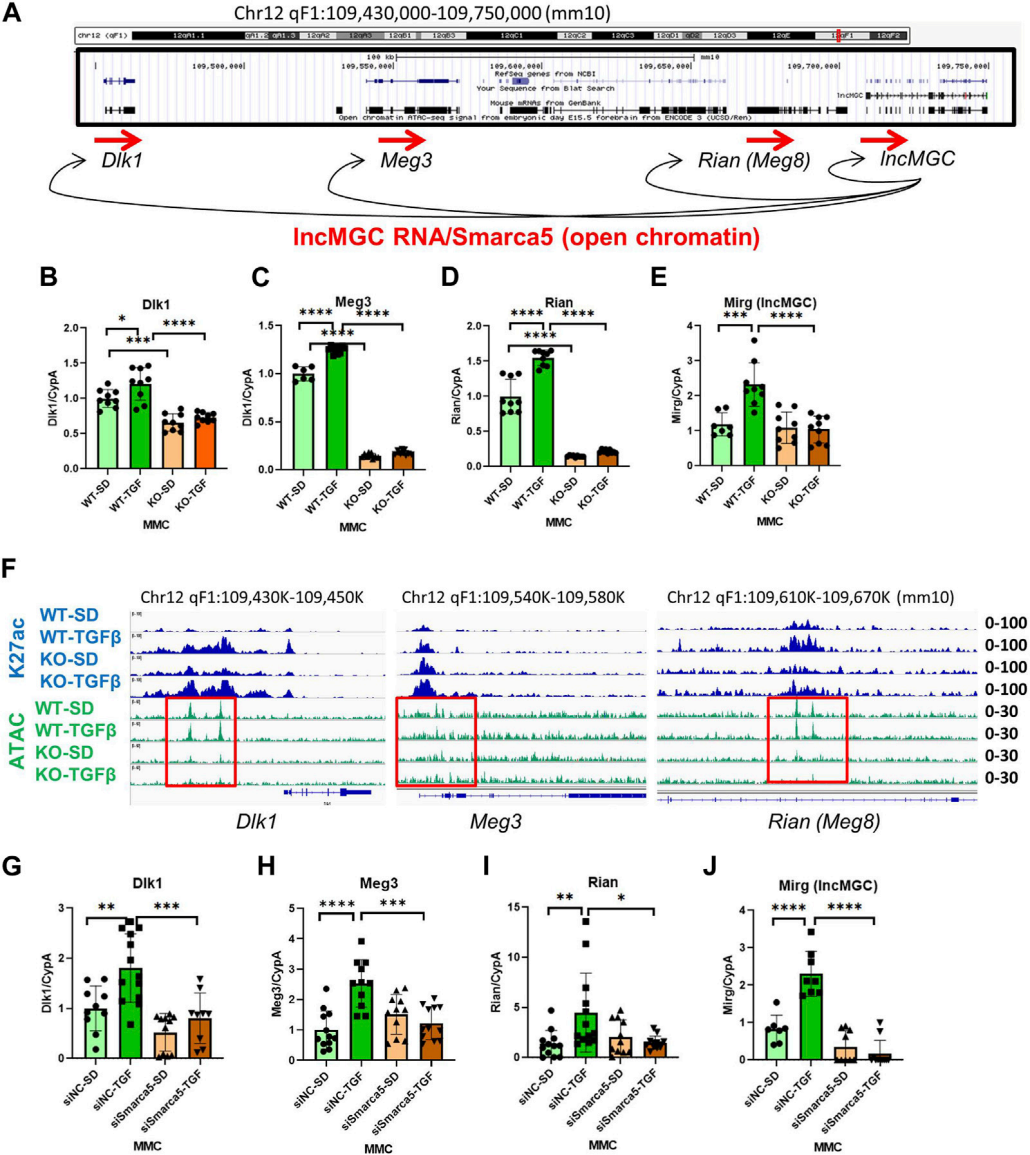
Effects of lncMGC deletion and SMARCA5 siRNA (siSMARCA5) on lncMGC neighboring genes. **(A)** Genomic structure of the Dlk1-lncMGC region and a proposed model of regulation of neighboring genes by lncMGC RNA through SMARCA5. **(B–E)** Expression levels (RT-qPCR) of lncMGC neighboring genes (*Dlk1, Meg3, Rian,* and *Mirg*) in KO5 MMC compared to WT MMC. (F) ATAC-seq and H3K27 ChIP-seq tracks in WT and lncMGC KO5 MMC showed changes at the TSS of lncMGC in KO5 MMC compared to WT MMC in control and TGF-β-treated conditions. ATAC-peaks at the TSS of lncMGC adjacent transcripts (*Dlk1, Meg2,* and *Rian*) are shown in KO5 MMC compared to WT MMC in control conditions and TGF-β-treated conditions. The positions of ATAC-peaks (green) are the same as those of H3K27ac (blue), suggesting those sites are critical for the expression of these genes. **(G–J)** Effects of siSMARCA5 in WT MMC on *Dlk1* **(G)**, *Meg3* **(H)**, *Rian* **(I)**, and *Mirg* **(J)**. Data are shown as the mean of three independent experiments calculated from triplicate qPCRs. One-way ANOVA with *post hoc* Tukey’s test for multiple comparisons; ±SEM; *, *p* < 0.05; **, *p* < 0.01; ***, *p* < 0.001; and ****p < 0.0001.

Several transcripts are mapped close to the lncMGC locus, prompting a closer look at ATAC-peaks from *Dlk1* to *Rian* (Figure 7A). Interestingly, several adjacent genes *(Dlk1, Meg3, Rian, and Mirg)* were significantly decreased in KO5 MMC compared to WT MMC (Figures 7B–E). Also, ATAC-peaks at these neighboring transcripts (*Dlk1, Meg3,* and *Rian*) were reduced in KO5 MMC compared to WT MMC, regardless of TGF-β treatment (Figure 7F). These results suggest that lncMGC KO affects the chromatin structures of neighboring genes. Similar to the lncMGC promoter, the chromatin of neighboring regions of lncMGC was open in WT MMC before TGF-β treatment but closed in KO5 MMC even after TGF-β treatment. In addition, the expression of neighboring genes (*Dlk1, Meg3, Rian,* and *Mirg*) was significantly decreased in WT MMC treated with siSMARCA5, although they were significantly increased by TGF-β in siNC control-treated cell (Figures 7G–J). These results support that SMARCA5 (opening chromatin) is essential to induce TGF-β-regulated genes including neighboring genes, and that its interaction with lncMGC RNA may enhance the opening of chromatin and the expression of neighboring genes and its promoter (Figure 7A). Hi-C and Virtual 4C data (http://3dgenome.fsm.northwestern.edu/virtual4c.php) support the interactions from the human DLK1 region to the lncMGC region (Supplementary Figure S11A, S11B). Overall, these results demonstrate the regulation of neighboring genes by lncMGC through DNA/DNA and DNA/RNA interactions.

### 3.7 Motif analysis

**FIGURE 8:**
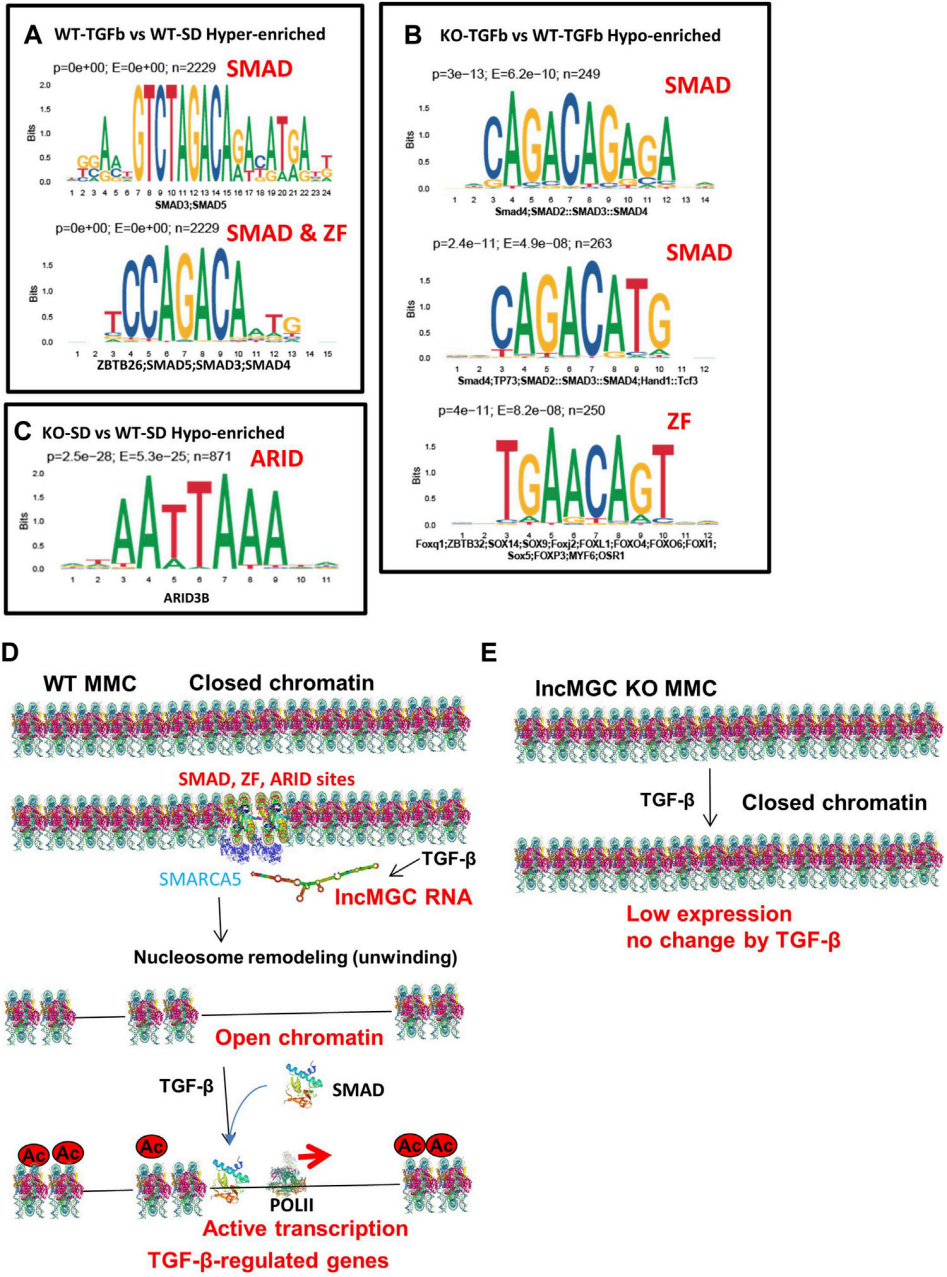
Motif analyses and proposed mechanism by which lncMGC and SMARCA5 regulate DKD-related genes by TGF-β. **(A–C)** Motif analysis (based on differential ATAC-peaks) of WT-TGFb *vs.* WT-SD Hyper (*p* < 0.0001 and enrichment >2) **(A)**, and KO-TGFb *vs.* WT-TGFb Hypo (*p* < 0.0001 and enrichment >2) **(B)** and KO-SD *vs.* WT-SD hypo (*p* < 0.0001 and enrichment >2) **(C)**. **(D)** Proposed mechanisms of gene regulation by lncMGC mediated by SMARCA5. The promoters of TGF-β-regulated genes are open through Smad and ZF ARID sites even before TGF-β treatment. TGF-β-regulated genes are upregulated by the recruitment of Smad at open chromatin regions. **(E)** In the absence of lncMGC, the promoters of TGF-β-regulated genes are closed even after TGF-β treatment, and their expression is not increased even with promoter acetylation or TGF-β treatment. Interaction of lncMGC RNA with nucleosome remodelers can enhance the gene expression through the opening of chromatin at the promoter regions of TGF-β-regulated genes.

As shown previously, not all DKD-related genes were regulated by lncMGC. To investigate the differences between regulated and unregulated lncMGC genes, we explored enriched motifs at ATAC-peaks and differentially expressed genes. Based on differential ATAC-peaks (Figures 8A–C), Smad binding sites were significantly higher in WT MMC treated with TGF-β (Figure 8A), in line with the fact that Smad proteins transduce TGF-β signaling (Massague et al., 2005; Kato and Natarajan, 2014; Meng et al., 2016; Kato and Natarajan, 2019). Smad motifs were also lower in KO5-TGF compared to WT-TGF (Figure 8B). This is in line with our observation that the promoter regions of key TGF-β-regulated genes were closed in KO5 MMC. Zinc finger (ZF), ZBTB26 (ZF and BTB domain 26), sites were increased in WT-TGF compared to WT-SD (Figure 8A). ZF, ZBTB32 (ZF and BTB domain 32), sites were less enriched in KO-TGF compared to WT-TGF MMC (Figure 8B). Interestingly, AT-rich sequences (ARID3b) were also lower in KO-SD *vs.* WT-SD (Figure 8C). Nucleosome remodelers have AT-rich interacting domains (ARIDs) (Becker and Hörz, 2002) and, thus, may be recruited to AT-rich sequences at ATAC-peaks. Based on DEG analysis (Supplementary Tables S1–S4), ZF sites and Smad motifs were enriched in certain genes that were increased in WT MMC treated with TGF-β (Supplementary Table S1). Interestingly, ZF motifs were also enriched in the downregulated genes in KO5 MMC compared to WT MMC (both in untreated and TGF-β conditions) (Supplementary Table S2–S4). These data suggest that the lncMGC-SMARCA5 complex may be preferentially recruited to the ZF, ARID, and Smad sites and facilitate chromatin opening and expression of the associated TGF-β-regulated genes (Figure 8D).

### 3.8 ZF and ARID motif in the lncMGC DNA sequence

We also examined transcription factor (TF) motifs in the lncMGC DNA sequence and found ZF motifs (GGT/GGG/CCC/ACC repeats) and AT-rich motifs in both human and mouse lncMGC genes (Supplementary Figures S12A, S12B). Interestingly, the sequence that was deleted in lncMGC-KO5 *vs.* WT contains such important ZF and AT-rich motifs (Supplementary Figures S12C–S12E). This further supports the notion that such motifs in the lncMGC may be critical for recruiting SMARCA5 to specific binding sites (ZF and ARID) on target genes.

## 4 Discussion

Herein, we identified nucleosome remodelers, including SMARCA5, as lncMGC-interacting proteins. These chromatin remodeling factors belong to the SWI/SNF protein complex, which has an ATPase/helicase domain and participates in unwinding DNA, RNA, and nucleosomes to relax chromatin (Clapier et al., 2017). As our data showed lncMGC interacts with several nucleosome remodelers, lncMGC may regulate the chromatin structure through the SMARC family proteins (and other remodelers) to enhance gene expression. Since open chromatin can maintain the active expression of DKD-related genes (Kato et al., 2013; Kato and Natarajan, 2019), nucleosome remodelers and their interactions with lncRNAs such as lncMGC may play important roles in the regulation of such genes in the pathogenesis of DKD. STRING DB also identified nucleosome remodelers as mouse lncMGC RNA-interacting proteins. The function of lncMGC RNA may be conserved between species from human to mouse.

H3K27ac and SMARCA5 enrichments were increased at the Smad site and initiator site (INR, TSS) of the lncMGC promoter in WT MMC treated with TGF-β but unchanged in KO5 MMC (Figures 6B–E). H3K27ac at the CHOP site in KO5 MMC was lower compared to WT MMC. These results suggest that lncMGC regulates its promoter. Promoter SMARCA5 enrichment was increased in WT MMC treated with TGF-β and unchanged in TGF-β-treated KO5 MMC even after TGF-β treatment (Figures 6F–H). siSMARCA5 reduced TGF-β induced expression of lncMGC and miR-379 and also H3K27ac levels at the lncMGC promoter Smad site (Figures 6I–K), suggesting that SMARCA5 is recruited to the lncMGC promoter through the interaction with lncMGC RNA to promote chromatin structure opening (Figure 8D).

Interestingly, ATAC-seq showed that the promoter region of lncMGC was constitutively open in WT MMC but closed in KO5 MMC, even after treatment with TGF-β (Figure 6A and Supplementary Figure S10). Therefore, an open chromatin is likely critical for the expression of lncMGC, and TGF-β may increase H3K27ac at lncMGC Smad and INR sites through SMARCA5 recruitment. It is reasonable to conjecture that SMARCA5 unwinds histone structures at the promoter region of lncMGC. Smad and INR sites may create a loop structure at the lncMGC promoter to enhance chromatin remodeling and the expression of lncMGC (autoregulation). Because KO5 MMC did not show increases of H3K27ac and SMARCA5 enrichment by TGF-β treatment, lncMGC RNA may play a critical role to unwind histone structures by recruiting SMARCA5 in *cis* (at lncMGC loci). On the other hand, because the 5’ region of lncMGC was also deleted in lncMGC-KO5 MMC, we cannot totally rule out the possibility that the genomic change (deletion) in lncMGC-KO5 MMC caused the reduced expression of lncMGC.

ATAC-seq also demonstrated that the chromatin structure of transcripts adjacent to lncMGC (*Dlk1, Meg3,* and *Rian*) were similarly changed; that is, peaks were reduced in KO5 MMC compared to WT MMC in control and TGF-β-treated conditions (Figure 7). Publicly available HiC data demonstrated interactions from *DLK1* to the lncMGC region (Supplementary Figure S11), perhaps through DNA/DNA and DNA/RNA interactions. More interestingly, ATAC-seq showed that numerous TGF-β-responsive genes were regulated in the same fashion. Their expression was reduced in lncMGC KO or SMARCA5 knockdown (KD with siRNA) MMC and their chromatin state was open prior to TGF-β treatment in WT MMC but closed in TGF-β-treated KO5 MMC. This suggests that lncMGC–SMARCA5 interaction facilitates chromatin opening and promotes the expression of TGF-β-responsive genes and effects are lost in lncMGC KO cells (Figures 8D,E). Other DKD-related genes such as *Col4a3, Col4a4, Ucp2, Nkd2,* and *Nox4* were also downregulated in lncMGC KO MMC or SMARCA5 KD MMC (Figure 3). Such genome-wide effects on gene expression (especially in pro-fibrotic genes) were also observed by the antisense oligonucleotide (ASO) inhibitor (GapmeR) of lncMGC in our previous study (Kato et al., 2016), although the results in the current KO5 MMC showed clearer conclusion possibly because ASO cannot shut down gene expression completely. Therefore, lncMGC may have widespread functions in DKD-related gene expression through interactions with nucleosome remodelers such as SMARCA5 (Figures 8D, E).

Motif analysis suggested that Smad, ZF, and AT-rich sites were enriched in ATAC-seq peaks and DEGs between WT and KO MMC (Figures 8A–C, Supplementary Table S1–S4). This could be predicted given that Smad proteins are major canonical signal transducers of TGF-β signaling (Massague et al., 2005; Kato and Natarajan, 2014; Meng et al., 2016; Kato and Natarajan, 2019). Nucleosome remodelers have ARID domains (Becker and Hörz, 2002) and may be recruited to AT-rich sequences at the ATAC-peaks. Interestingly, the sequence that was deleted in lncMGC to obtain lncMGC-KO5 MMC included such ZF and ARID consensus sequences. Thus, the lncMGC-SMARCA5 complex may be preferentially recruited to such specific sites (Smad, ZF, and ARID) to enhance chromatin opening and the expression of TGF-β-regulated genes (Figure 8D). ZF and ARID sequences in lncMGC may, in turn, also assist in the recruitment of lncMGC-SMARCA5 to such specific sites through DNA/RNA or DNA/DNA interactions. As reported previously, the abundance of lncMGC RNA is relatively low (∼six copies/cell even in the TGF-β or HG condition) (Kato et al., 2016). However, a significant increase of lncMGC RNA by TGF-β may be enough for the genome-wide effects, and such low abundance may also be important for the regulation of gene expression at specific sites because the specificity might be lost if it is in abundance.

Interestingly, we found that overlaps of ATAC-peaks and CpG islands in upenriched ATAC peak sites in KO MMC (KO > WT) were significantly greater than those in downenriched ATAC-peak sites (KO < WT) in KO MMC (Supplementary Figure S13A). These results suggest that lncMGC may also mediate gene expression through CpG islands and ZF transcription factors and that lncMGC may preferentially open chromatin at non-CpG island regions but close chromatin at CpG island regions (Supplementary Figure S13B). The reanalysis of publicly available metadata (N = 33605) on DNA methylation associated with kidney function and damage suggested a negative association of eGFR with DNA methylation at key CpGs in the lncMGC loci (Supplementary Table S5) (Schlosser et al., 2021). The same report showed that the top two eGFR-associated CpGs were at the genes encoding the ZF proteins ZNF788 and JAZF1 (Schlosser et al., 2021). These data also support our results showing ZF sites were enriched at DEGs between WT and KO MMC (Supplementary Table S1–S4). Moreover, we observed KRAB-associated protein 1 (KAP1) was also an lncMGC-interacting protein (Figure 1C). The affinity of lncMGC to KAP1 is almost the same as that reported for non-coding 7SK RNA (McNamara et al., 2016) (Figure 1D). KAP1 interacts with KRAB-ZFPs and is known to be a transcriptional corepressor associated with DNA methylation and imprinting (Schultz et al., 2002; Groner et al., 2010; Yang et al., 2017). Hence, lncMGC-RNA-KAP1-ZFP may play a putative role in maintaining repressed chromatin structures in the regulation of DKD-related and TGF-β-regulated genes downregulated by lncMGC. This also supports the possibility of autoregulation of the lncMGC region by DNA methylation and KAP1 associated with lncMGC RNA, although more work is needed to verify these mechanisms.

In summary, using an integrative Omics coupled with mechanistic studies, we found that lncMGC regulates TGF-β-responsive genes, including fibrotic, inflammatory, and mitochondrial metabolic genes, via nucleosome remodelers which alter the chromatin status to a more accessible configuration. As the inhibition of lncMGC ameliorated DKD-related features in mouse models (Kato et al., 2016), targeting lncRNA-interacting nucleosome remodelers such as SMARCA5 may also be tested as potential new therapeutics for DKD in the future.

## Data Availability

All sequencing datasets generated in this study have been deposited into NCBI Gene Expression Omnibus repository, GEO (https://www.ncbi.nlm.nih.gov/geo/) with GEO numbers, GSE229686, GSE229687, GSE229688, GSE229689.
