# Supplementary material for: Long non-coding RNA lncMGC mediates the expression of TGF-β-induced genes in renal cells via nucleosome remodelers
Source: Front Mol Biosci. 2023 May 30;10:1204124. doi: 10.3389/fmolb.2023.1204124 (PMC10266347; doi:10.3389/fmolb.2023.1204124)
Supplement: Supplementary file 1 [file DataSheet1.pdf]

Supplementary Information

Long noncoding RNA lncMGC mediates the expression of TGF-β-induced genes in renal cells via nucleosome remodelers

Mitsuo Kato[1], Zhuo Chen[1], Sadhan Das[1,2], Xiwei Wu[3], Jinhui Wang[3], Arthur Li[3], Wei Chen[3], Walter Tsark[4], Ragadeepthi Tunduguru[1], Linda Lanting[1], Mei Wang[1], Roger Moore[5], Markus Kalkum[5], Maryam Abdollahi[1], and Rama Natarajan[1]

[1]Department of Diabetes Complications and Metabolism, Arthur Riggs Diabetes & Metabolism Research Institute, Beckman Research Institute of City of Hope, 1500 E. Duarte Rd, Duarte, CA 91010, USA, [2]Department of Biological Sciences, Indian Institute of Science Education and Research (IISER) Mohali, Knowledge City, Sector 81, SAS Nagar, Mohali, Punjab 140306, India, [3]Integrative Genomics Core, Beckman Research Institute of City of Hope, 1500 E. Duarte Rd, Duarte, CA 91010, USA. [4]Transgenic Mouse Facility, Center for Comparative Medicine, Beckman Research Institute of City of Hope, 1500 E. Duarte Rd, Duarte, CA 91010, USA. [5]Department of Immunology & Theranostics, Arthur Riggs Diabetes & Metabolism Research Institute, Beckman Research Institute of City of Hope, 1500 E. Duarte Rd, Duarte, CA 91010, USA.

Correspondence:
Rama Natarajan, Ph.D.
Mitsuo Kato, PhD

Email : RNatarajan@coh.org
Email : mkato@coh.org

Department of Diabetes Complications and Metabolism
Arthur Riggs Diabetes & Metabolism Research Institute
Beckman Research Institute of the City of Hope
1500 East Duarte Road, Duarte, CA 91010
Tel: 626-218-2289, 626-218-3996
Fax: 626-301-8136

Suppl.Fig.1

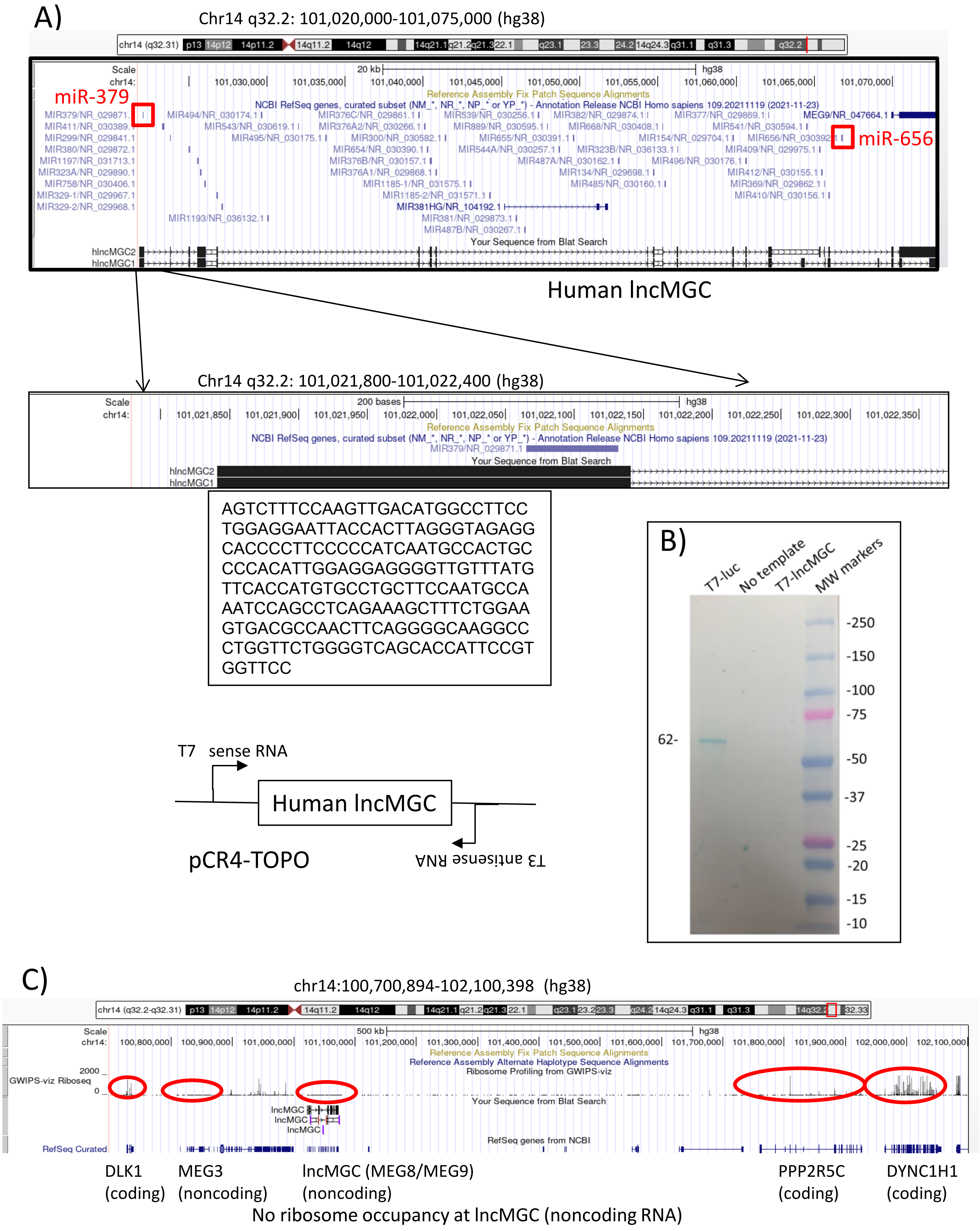
A)
Chr14 q32.2: 101,020,000-101,075,000 (hg38)
miR-379
miR-656
NCBI RefSeq genes, curated subset (NM_*, NR_*, NP_* or YP_*) - Annotation Release NCBI Homo sapiens 109.20211119 (2021-11-23)
Reference Assembly Fix Patch Sequence Alignments
Your Sequence from Blat Search
MEG9/NR_047664.1
MIR381HG/NR_104192.1
hlncMGC2
hlncMGC1
Human lncMGC
Chr14 q32.2: 101,021,800-101,022,400 (hg38)
MIR379/NR_029871.1
hlncMGC2
hlncMGC1
AGTCTTTCCAAGTTGACATGGCCTTCC
TGGAGGAATTACCACTTAGGGTAGAGG
CACCCCTTCCCCCATCAATGCCACTGC
CCCACATTGGAGGAGGGGTTGTTTATG
TTCACCATGTGCCTGCTTCCAATGCCA
AATCCAGCCTCAGAAAGCTTTCTGGAA
GTGACGCCAACTTCAGGGGCAAGGCC
CTGGTTCTGGGGTCAGCACCATTCCGT
GGTTCC
T7 sense RNA
Human lncMGC
pCR4-TOPO
T3 antisense RNA
B)
T7-luc
No template
T7-lncMGC
MW markers
62-
-250
-150
-100
-75
-50
-37
-25
-20
-15
-10
C)
chr14:100,700,894-102,100,398 (hg38)
GWIPS-viz Riboseq
Ribosome Profiling from GWIPS-viz
Your Sequence from Blat Search
RefSeq genes from NCBI
RefSeq Curated
lncMGC
DLK1 (coding)
MEG3 (noncoding)
lncMGC (MEG8/MEG9) (noncoding)
PPP2R5C (coding)
DYNC1H1 (coding)
No ribosome occupancy at lncMGC (noncoding RNA)

Supplementary Figure 1. Human lncMGC and screening of human lncMGC-interacting proteins. A, Genomic structure of human lncMGC (hlncMGC; GenBank MW802746 & MW802747) which covers miR-379 miRNA cluster from miR-379 (most 5') to miR-656 (most 3') on human Chromosome 14q32.2. hlncMGC (GenBank MW802745) was cloned into the pCR4-TOPO expression vector (ThermoFisher Scientific) by using T7 or T3 promoter in the plasmid to express sense or antisense hlncMGC RNA. B, No protein from hlncMGC RNA was detected (T7-lncMGC) although luciferase protein was detected from luc-RNA (T7-luc, positive control). C, Ribosome occupancy (GWIPS-viz, https://gwips.ucc.ie/) at the lncMGC locus . Very low or no ribosome occupancy was detected at lncMGC and also other noncoding RNA regions (MEG3) although significant ribosome occupancy was detected at coding regions (DLK1, PPP2R5C and DYNC1H1).

Suppl. Fig.2

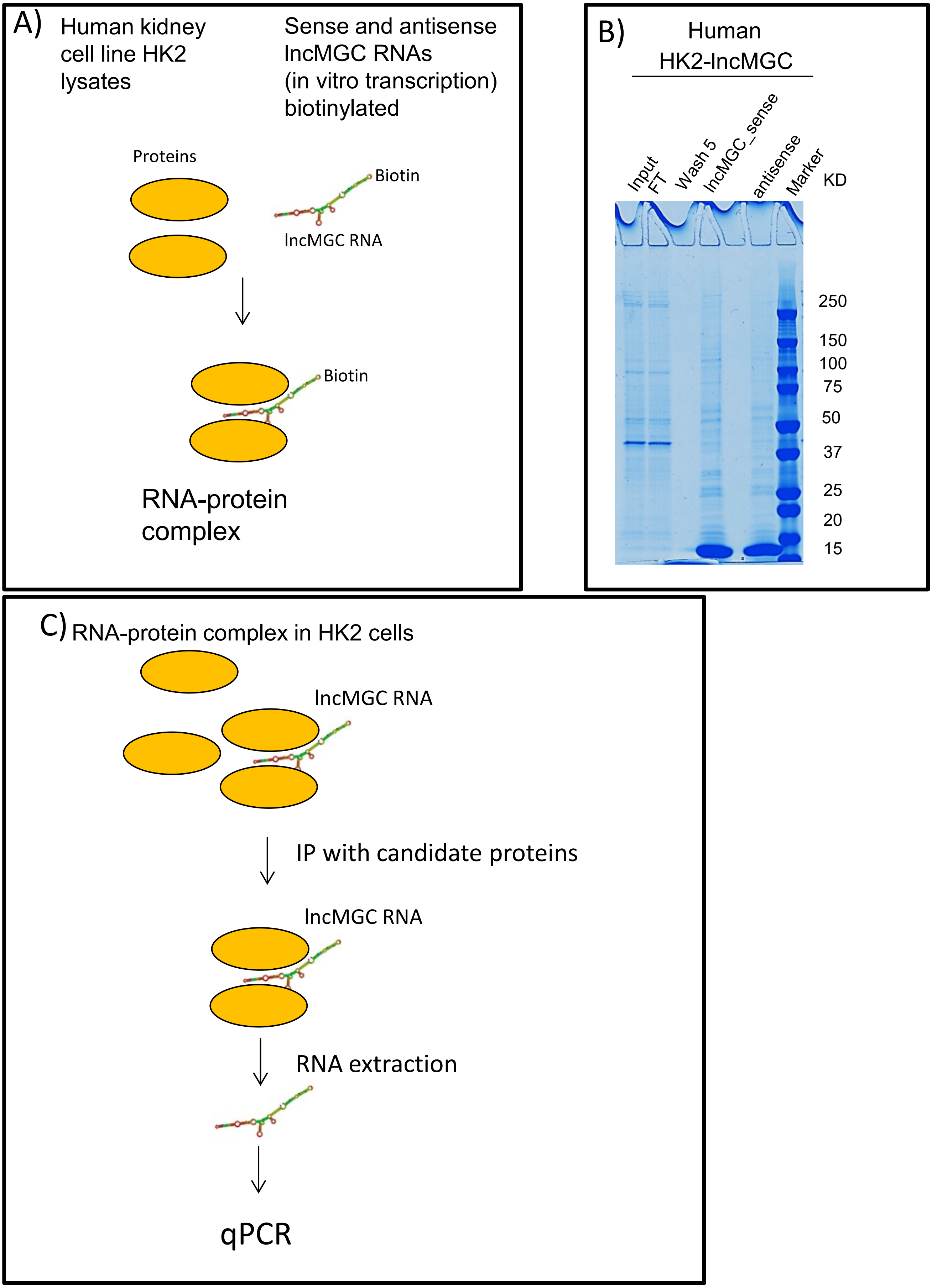
A)
Human kidney cell line HK2 lysates
Sense and antisense lncMGC RNAs (in vitro transcription) biotinylated
Proteins
Biotin
lncMGC RNA
Biotin
RNA-protein complex
B)
Human
HK2-lncMGC
Input
FT
Wash 5
lncMGC_sense
antisense
Marker
KD
250
150
100
75
50
37
25
20
15
C) RNA-protein complex in HK2 cells
lncMGC RNA
IP with candidate proteins
lncMGC RNA
RNA extraction
qPCR

Supplementary Figure 2. Identification of lncMGC-interacting proteins by Mass spec. A, *In vitro* transcribed biotinylated hlncMGC RNAs (sense and antisense) were mixed with human kidney cell (HK2) lysate and RNA-protein complexes were isolated. B, Isolated proteins were separated in SDS-PAGE gel and subjected to Mass Spectrometry. C, Confirmation of lncMGC interaction with candidate proteins. Interaction of lncMGC and candidate proteins in HK-2 cells was confirmed by immunoprecipitation (IP) with antibodies to the candidate interacting proteins followed by RT-qPCR.

Suppl.Fig.3

A)

Mouse lncMGC

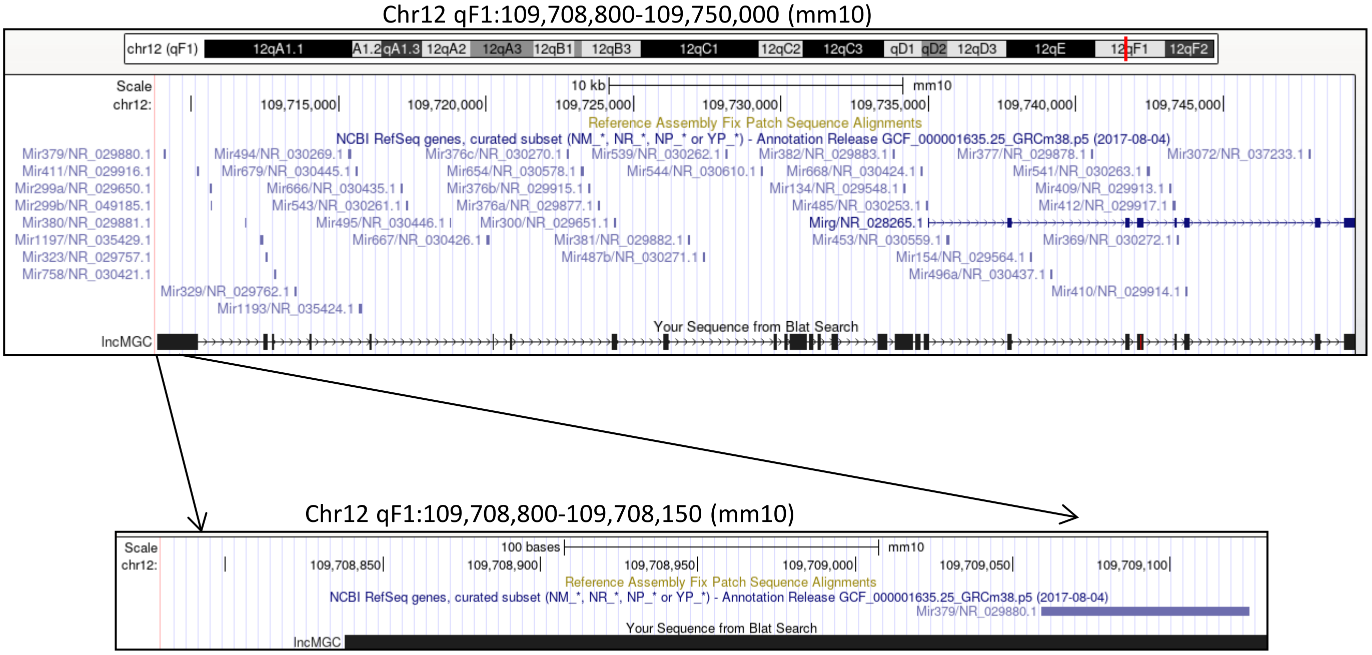

B)

ATTTTTCTGAGTTAGTGTGGCCTTCATCTGGTAATGT
ACTACCTGAGGGGGGAGGTGCCGCCTCTCTTTCAG
CACCGTGCAACCATTCAAGGAGGGTGTGTTGTTCAC
CACATCTGCTTCCCACTGCCAAATCAGGCCTCAGAA
AAGCTTTCTGGAAGTGACGCCAGCTTCAGGGACAA
GGCCCAAGTTTCTAGGGGTCAACACCGTTCCATGGT
TCCTG

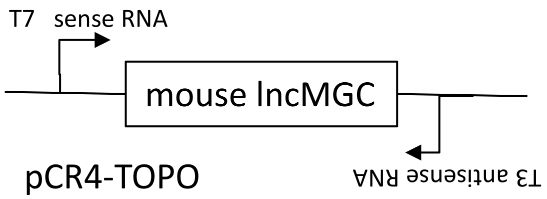

In vitro transcription

Supplementary Figure 3. Mouse lncMGC. A, Genomic structure of mouse lncMGC (lncMGC; GenBank MW802743, MW802744) which covers miR-379 miRNA cluster from miR-379 (most 5’) to miR-3072 (most 3’) on mouse Chromosome 12qF1. B, lncMGC (GenBank MW802744) was cloned into the expression vector pCR4-TOPO (ThermoFisher Scientific) to express sense or antisense lncMGC RNA.

Suppl. Fig.4

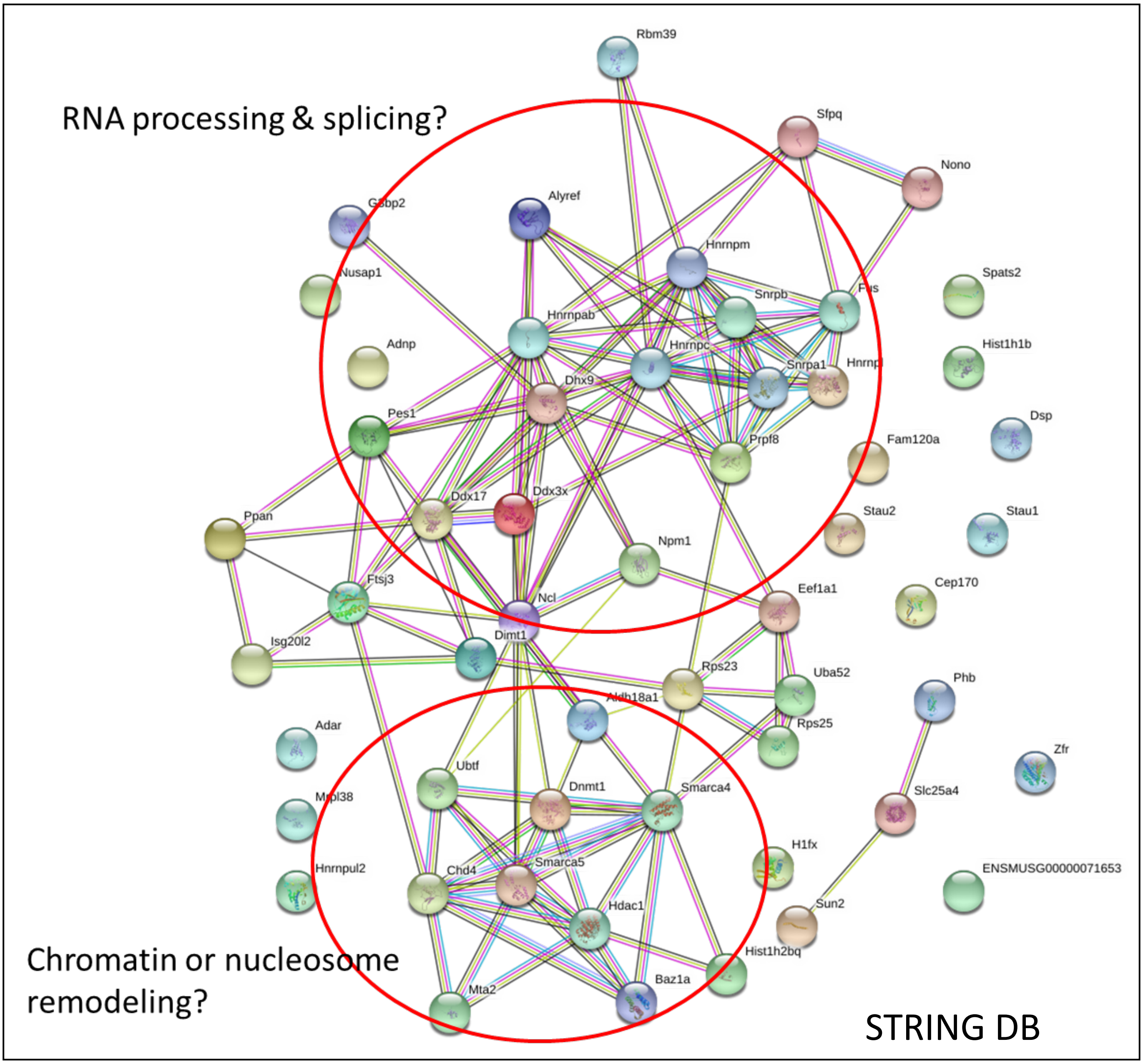

lncMGC interacting proteins in Mouse TCMK-1 cells

Supplementary Figure 4. Mouse lncMGC-interacting proteins. STRING DB analysis shows interaction of proteins with lncMGC in mouse kidney TCMK-1 cells identified by MS. Groups of RNA processing factors and nucleosome remodeling factors were identified. Smarca5 is in center of interaction of identified proteins.

Suppl. Fig.5

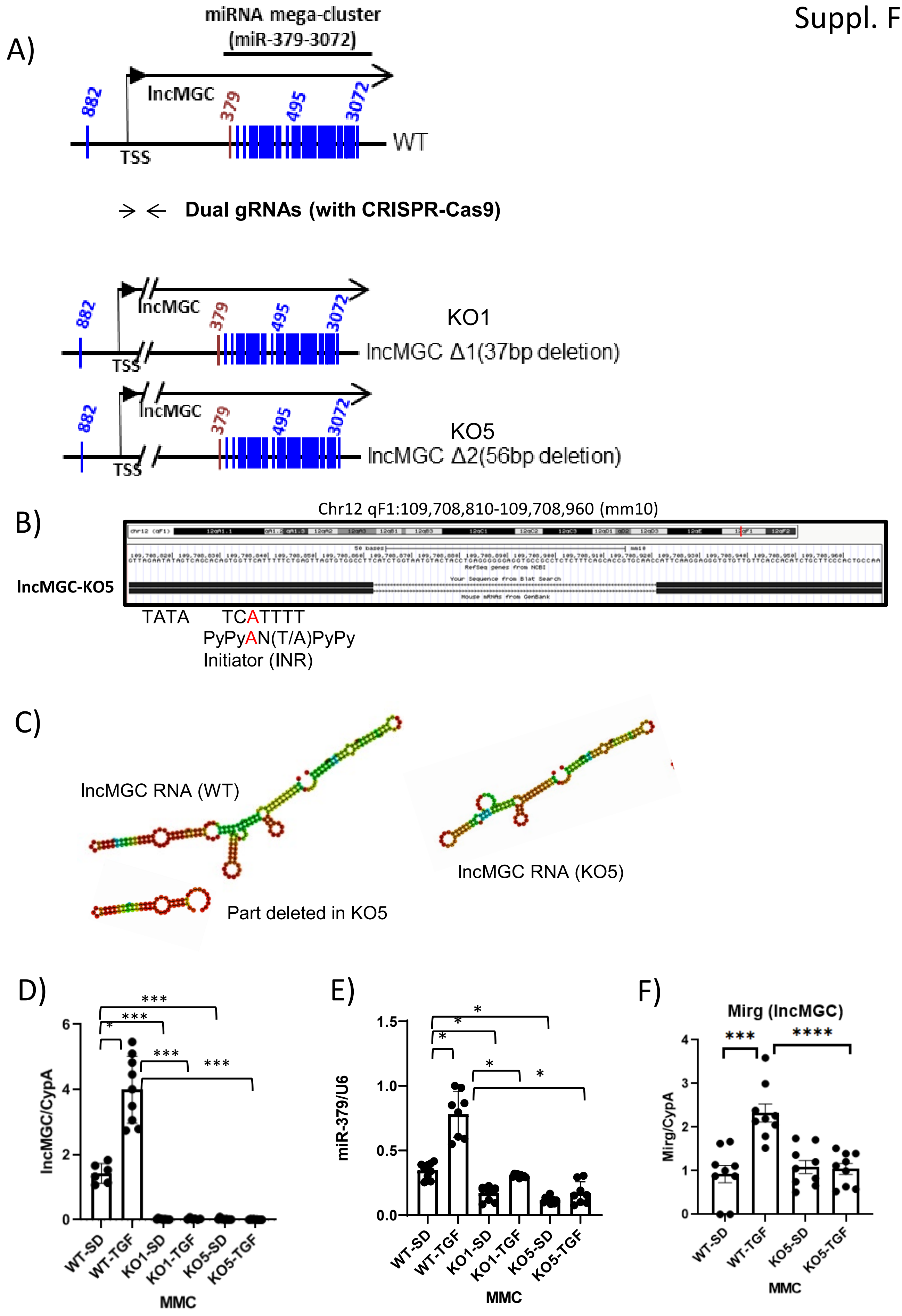

A)
miRNA mega-cluster
(miR-379-3072)
lncMGC
882
379
495
3072
TSS
WT
Dual gRNAs (with CRISPR-Cas9)
KO1
lncMGC Δ1(37bp deletion)
KO5
lncMGC Δ2(56bp deletion)
B)
Chr12 qF1:109,708,810-109,708,960 (mm10)
lncMGC-KO5
TATA
TCATTTT
PyPyAN(T/A)PyPy
Initiator (INR)
C)
lncMGC RNA (WT)
lncMGC RNA (KO5)
Part deleted in KO5
D)
lncMGC/CypA
WT-SD
WT-TGF
KO1-SD
KO1-TGF
KO5-SD
KO5-TGF
MMC
E)
miR-379/U6
MMC
F)
Mirg (lncMGC)
Mirg/CypA
MMC

Supplementary Figure 5. lncMGC KO mice. A, lncMGC knockout (KO) mice were created using the CRISPR-Cas9 system and dual gRNAs to delete the sequence around the transcription start site (TSS). B, Deletions at TSS were confirmed in KO mouse lines (KO1, 37 bp and KO5, 56 bp) by the chain termination method of Sanger sequencing. C, Secondary structures of lncMGC RNA including the deleted region in KO5 and KO5 lncMGC RNA (partially deleted) were predicted by the Vienna RNA Websuite (http://rna.tbi.univie.ac.at/). D-F) Changes in the expression of lncMGC, miR-379, and Mirg (a part of the 3' end of lncMGC) were confirmed in lncMGC-KO MMC by RT-qPCR. Data are shown as means of three triplicate PCRs. One-way ANOVA with Tukey's post hoc test for multiple comparisons; ±SEM; *, $P<0.05$; **, $P<0.01$; ***, $P<0.001$; ****$P<0.0001$.

Suppl. Fig.6

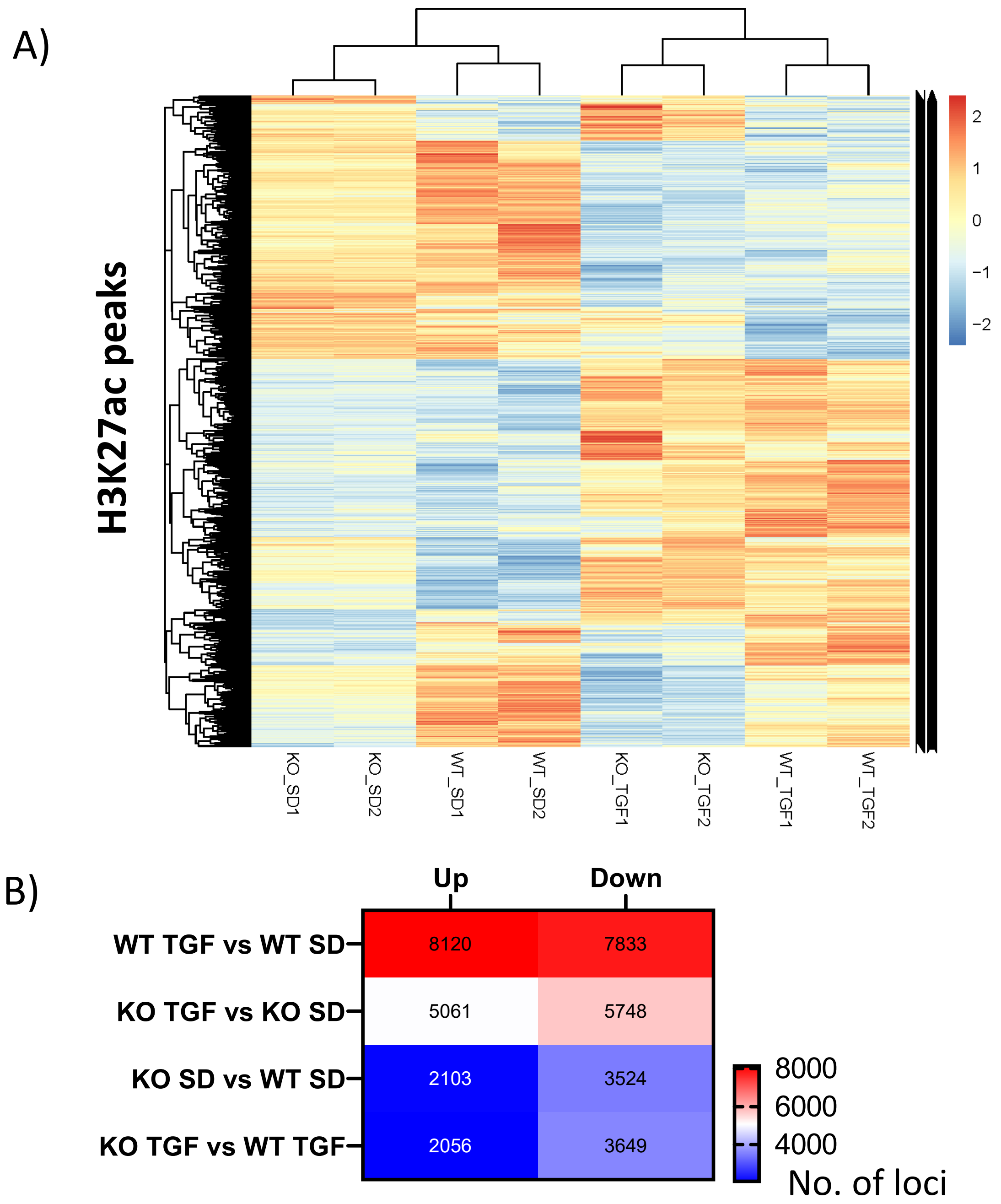

Supplementary Figure 6. H3K27ac ChIP-seq. A, Heat map of ChIP-seq data showing genome-wide changes in H3K27ac among samples from WT and KO under SD (control) and after TGF-β treatment (run in duplicates). B, Heat map showing the numbers of loci depicting increases in H3K27ac enrichment (Up) or decreases in H3K27ac enrichment (Down) in the indicated comparisons.

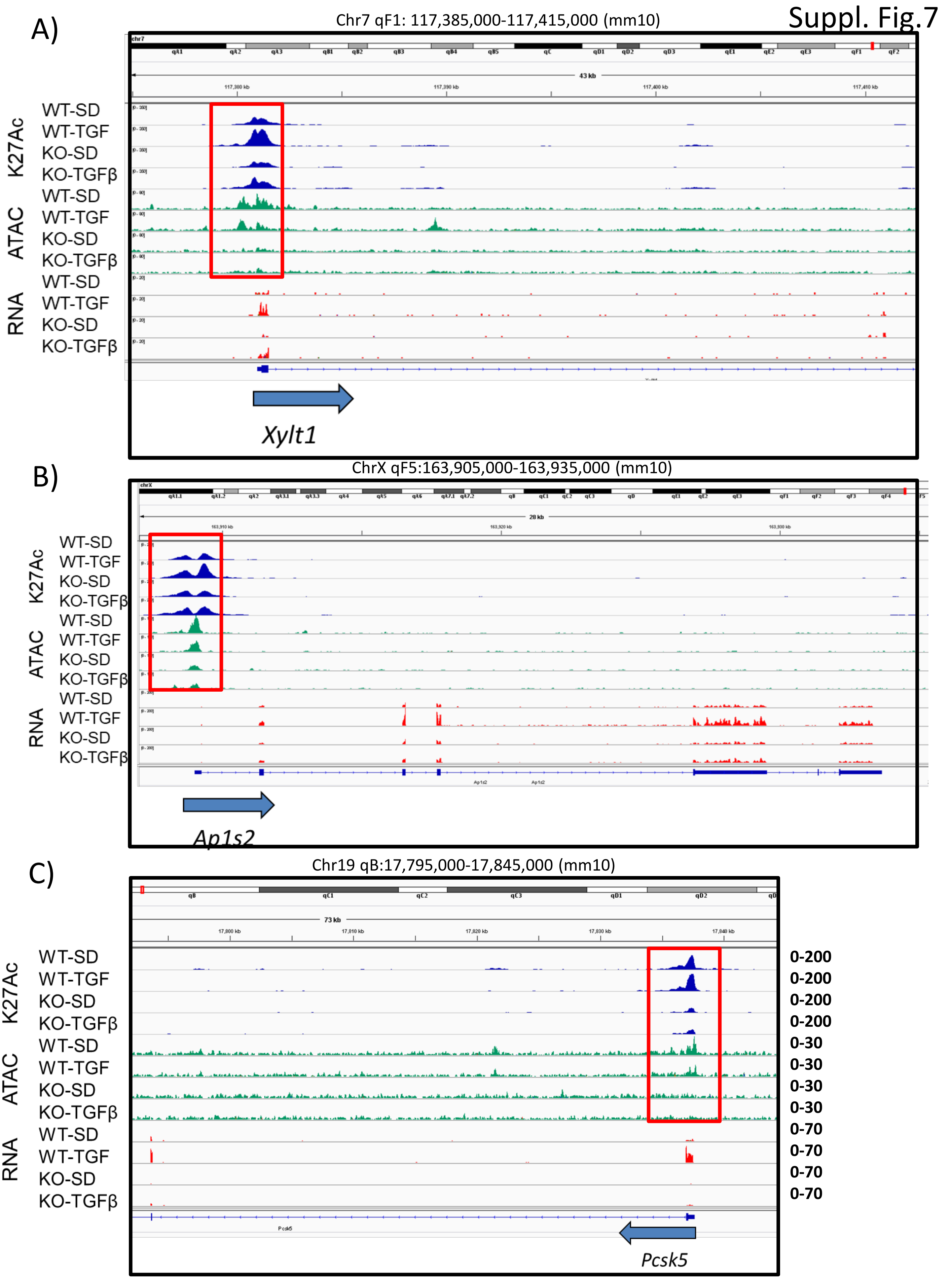
Suppl. Fig.7
A)
Chr7 qF1: 117,385,000-117,415,000 (mm10)
K27Ac
ATAC
RNA
WT-SD
WT-TGF
KO-SD
KO-TGFβ
WT-SD
WT-TGF
KO-SD
KO-TGFβ
WT-SD
WT-TGF
KO-SD
KO-TGFβ
Xylt1
B)
ChrX qF5:163,905,000-163,935,000 (mm10)
K27Ac
ATAC
RNA
WT-SD
WT-TGF
KO-SD
KO-TGFβ
WT-SD
WT-TGF
KO-SD
KO-TGFβ
WT-SD
WT-TGF
KO-SD
KO-TGFβ
Ap1s2
C)
Chr19 qB:17,795,000-17,845,000 (mm10)
K27Ac
ATAC
RNA
WT-SD
WT-TGF
KO-SD
KO-TGFβ
WT-SD
WT-TGF
KO-SD
KO-TGFβ
WT-SD
WT-TGF
KO-SD
KO-TGFβ
0-200
0-200
0-200
0-200
0-30
0-30
0-30
0-30
0-70
0-70
0-70
0-70
Pcsk5

Supplementary Figure 7. Combined analysis of H3K27ac ChIP-seq, ATAC-seq, and RNA-seq) at identified differentially expressed DKD-related genes *Xylt1 (A), Ap1s2(B)* and *Pcsk5 (C)* genes. Genomic tracks shown at the indicated gene loci. The expression of *Xylt1, Ap1s2* and *Pcsk5* genes was up-regulated by TGF-β in WT MMC but not in KO5 MMC (RNA–seq, red). The promoter regions of those genes are open before TGF-β treatment in WT MMC but closed in KO5 MMC even after TGF-β treatment (ATAC-seq, green). H3K27ac was increased by TGF-β treatment in both WT and KO5 MMC (blue).

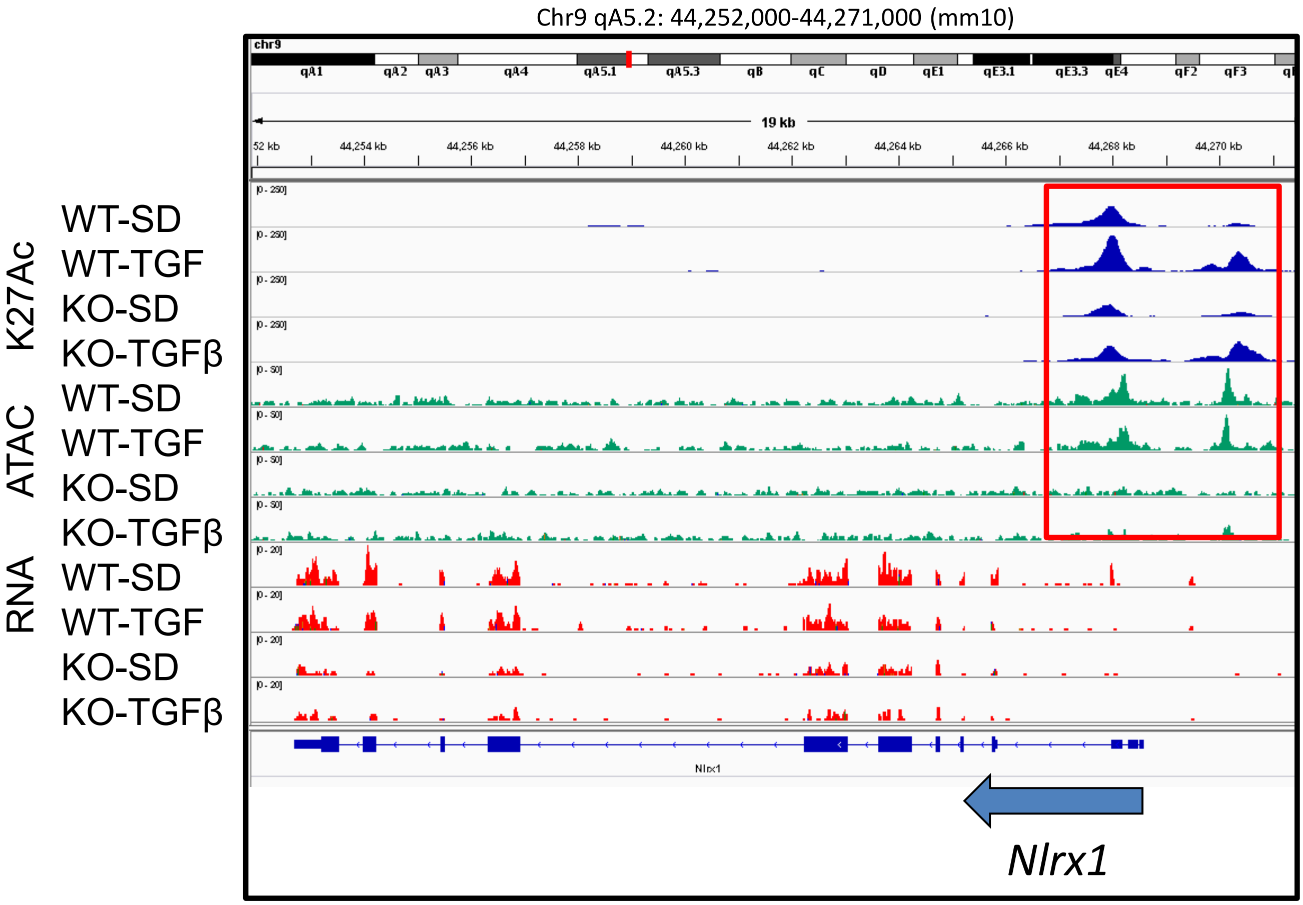

Supplementary Figure 8. Expression of *Nlrx1 (from Nlr* family of genes) in WT and KO5 MMC.

Combined analysis of (H3K27ac ChIP-seq, ATAC-seq, RNA-seq) at the *Nlrx1* gene. Genomic tracks shown. The expression of *Nlrx1*gene was not up-regulated by TGF-β in WT MMC but was decreased in KO5 MMC (RNA–seq, red) compared to WT MMC. The promoter regions of *Nlrx1* was open before TGF-β treatment in WT MMC but closed in KO5 MMC even after TGF-β treatment (ATAC-seq, green). H3K27ac was increased by TGF-β treatment in both WT and KO5 MMC (blue).

Suppl. Fig.9

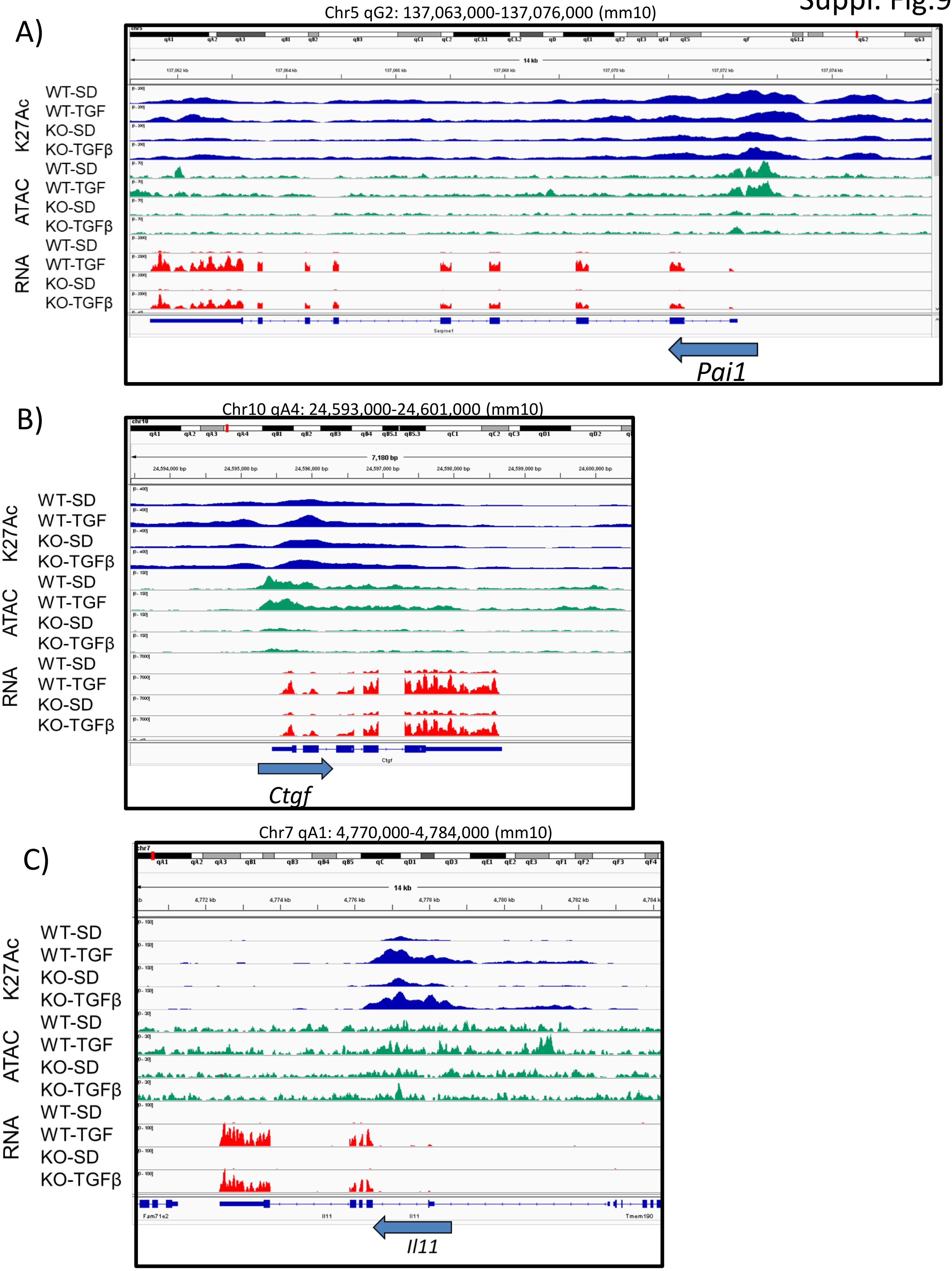
A)
Chr5 qG2: 137,063,000-137,076,000 (mm10)
K27Ac
WT-SD
WT-TGF
KO-SD
KO-TGFβ
ATAC
WT-SD
WT-TGF
KO-SD
KO-TGFβ
RNA
WT-SD
WT-TGF
KO-SD
KO-TGFβ
Pai1
B)
Chr10 qA4: 24,593,000-24,601,000 (mm10)
K27Ac
WT-SD
WT-TGF
KO-SD
KO-TGFβ
ATAC
WT-SD
WT-TGF
KO-SD
KO-TGFβ
RNA
WT-SD
WT-TGF
KO-SD
KO-TGFβ
Ctgf
C)
Chr7 qA1: 4,770,000-4,784,000 (mm10)
K27Ac
WT-SD
WT-TGF
KO-SD
KO-TGFβ
ATAC
WT-SD
WT-TGF
KO-SD
KO-TGFβ
RNA
WT-SD
WT-TGF
KO-SD
KO-TGFβ
Il11

Supplementary Figure 9. Combined analysis (H3K27ac ChIP-seq, ATAC-seq, RNA-seq) at *Pai1(A), Ctgf* (B) and *Ii11* (C) genes in WT and KO5 MMC ± TGF-β treatment. Genomic tracks shown at the indicated gene loci.

Suppl. Fig.10

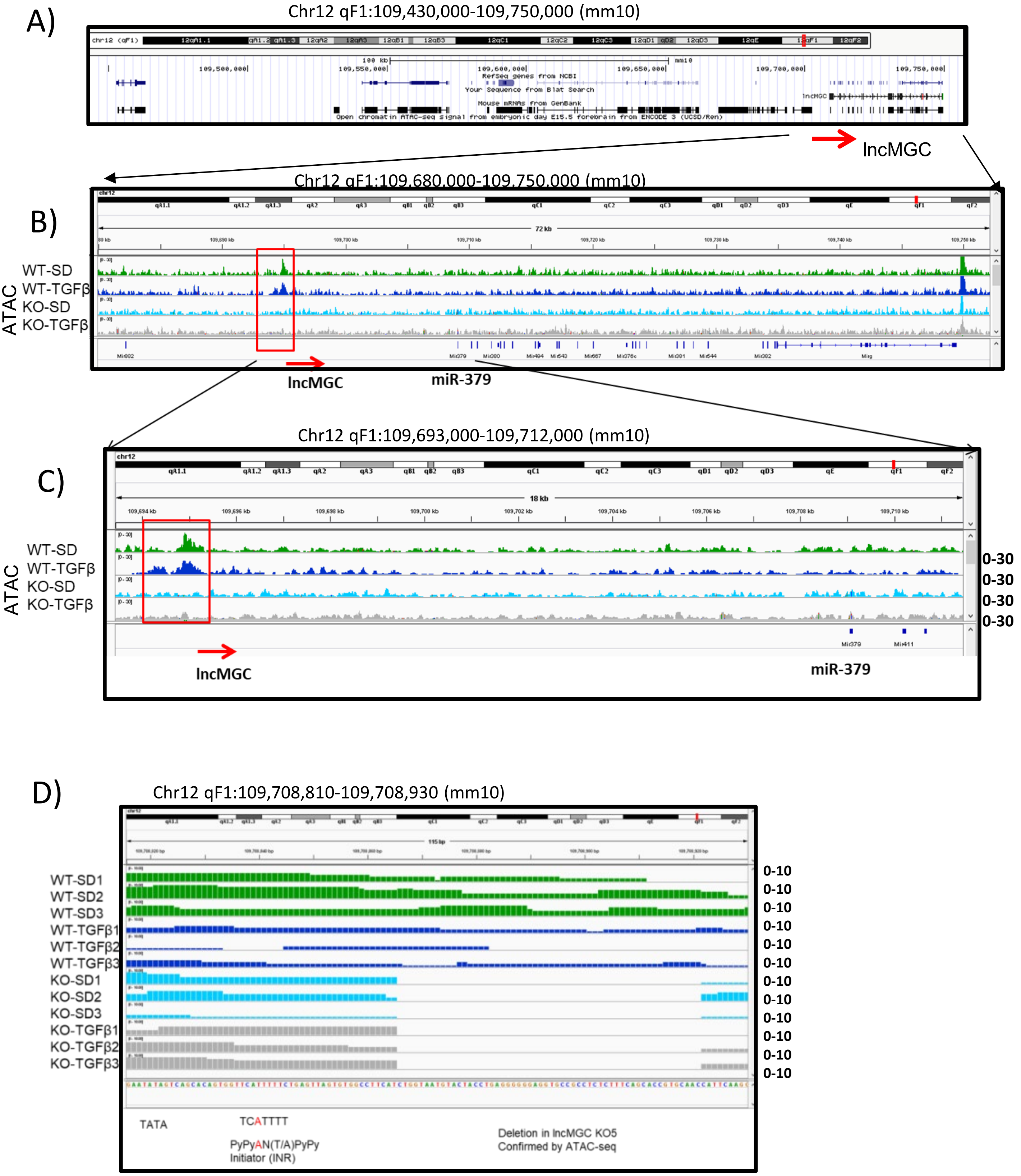
A)
Chr12 qF1:109,430,000-109,750,000 (mm10)
lncMGC
B)
Chr12 qF1:109,680,000-109,750,000 (mm10)
ATAC
WT-SD
WT-TGFβ
KO-SD
KO-TGFβ
lncMGC
miR-379
C)
Chr12 qF1:109,693,000-109,712,000 (mm10)
ATAC
WT-SD
WT-TGFβ
KO-SD
KO-TGFβ
0-30
0-30
0-30
0-30
lncMGC
miR-379
D)
Chr12 qF1:109,708,810-109,708,930 (mm10)
WT-SD1
WT-SD2
WT-SD3
WT-TGFβ1
WT-TGFβ2
WT-TGFβ3
KO-SD1
KO-SD2
KO-SD3
KO-TGFβ1
KO-TGFβ2
KO-TGFβ3
0-10
0-10
0-10
0-10
0-10
0-10
0-10
0-10
0-10
0-10
0-10
0-10
TATA
TCATTTT
PyPyAN(T/A)PyPy
Initiator (INR)
Deletion in lncMGC KO5
Confirmed by ATAC-seq

Supplementary Figure 10. ATAC-seq analysis around the lncMGC region. A, Genomic structure of mouse Dlk1- lncMGC region. B, ATAC-seq analysis of lncMGC region in WT and KO5 MMC ± TGF-β treatment (from TSS of lncMGC to 3' end of lncMGC). C, Close up figure of lncMGC region (from lncMGC to miR-379). Clear ATAC-peaks are detected at the promoter region of lncMGC in WT MMC but not in KO5 MMC. D, Higher resolution of ATAC-seq profile around the deleted region of lncMGC-KO5 MMC showing no detectable reads observed in KO5 MMC in the 56 bp deleted region relative to WT.

Suppl. Fig. 11

A) Hi-C data around human DLK1- lncMGC region

Chr14 q32.2: 99,00,000-103,000,000 (hg19)

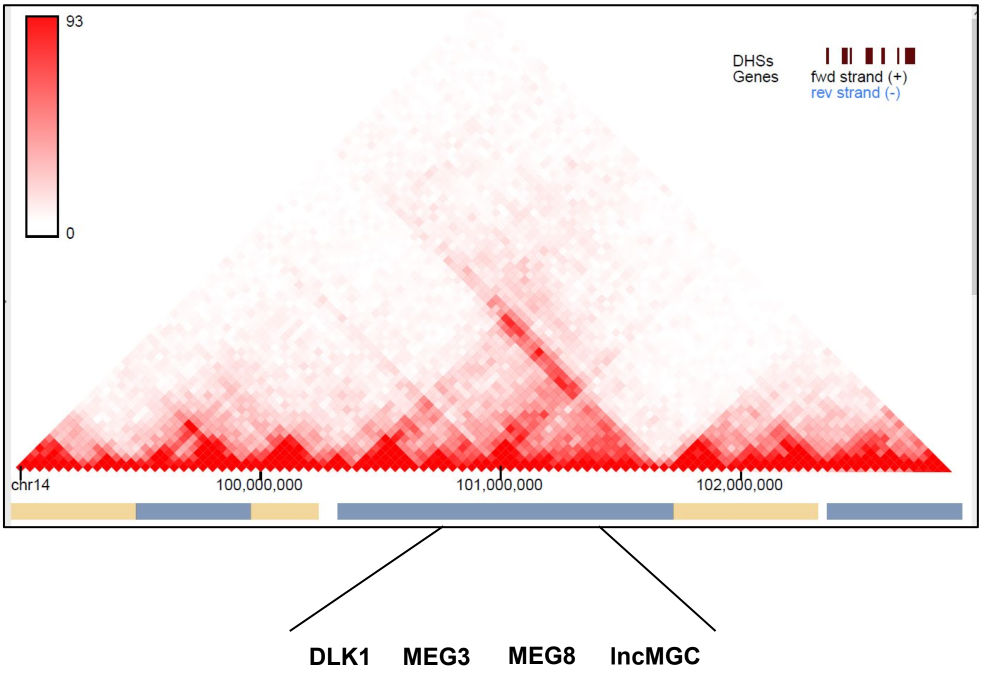

B) Virtual 4C data around human DLK1- lncMGC region

Chr14 q32.2: 101,100,000-101,550,000 (hg19)

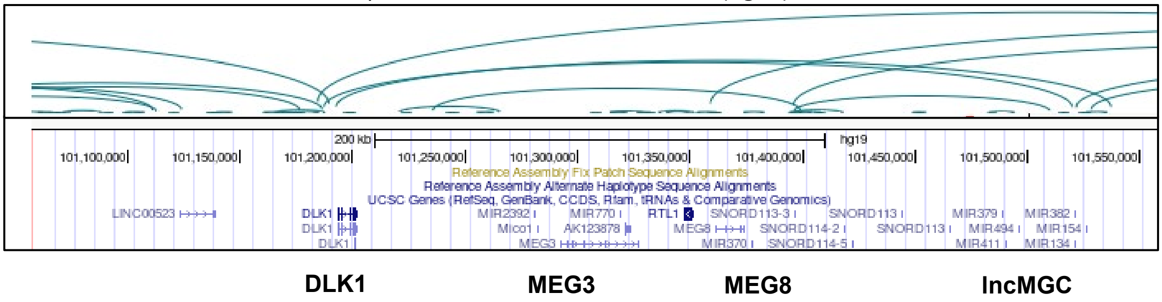

Supplementary Figure 11. Hi-C and virtual 4C data around human DLK1- lncMGC region. A, Hi-C data around human DLK1- lncMGC region depicted by 3D Genome Browser (http://3dgenome.fsm.northwestern.edu/virtual4c.php). Chr14 q32.2: 99,00,000-103,000,000 (hg19). B, Virtual 4C data around human DLK1- lncMGC region by 3D Genome Browser (http://3dgenome.fsm.northwestern.edu/virtual4c.php). Chr14 q32.2: 101,100,000-101,550,000 (hg19).

A)

Human lncMGC
AGTCTTTCCAAGTTGACATGGCCTTCCTGGAGGAATTACCACTTAGGGTAGAGGCACC
CCTTCCCCCATCAATGCCACTGCCCCACATTGGAGGAGGGGTTGTTTATGTTCACCAT
GTGCCTGCTTCCAATGCCAAATCCAGCCTCAGAAAGCTTTCTGGAAGTGACGCCAACT
TCAGGGGCAAGGCCCTGGTTCTGGGGTCAGCACCATTCCGTGGTTCC

B)

Mouse lncMGC
ATTTTTCTGAGTTAGTGTGGCCTTCATCTGGTAATGTACTACCTGAGGGGGGAGGTG
CCGCCTCTCTTTCAGCACCGTGCAACCATTCAAGGAGGGTGTGTTGTTCACCACATC
TGCTTCCCACTGCCAAATCAGGCCTCAGAAAGCTTTCTGGAAGTGACGCCAGCTTC
AGGGACAAGGCCCAAGTTTCTAGGGGTCAACACCGTTCCATGGTTCCTG

C)

Mouse lncMGC-KO5
ATTTTTCTGAGTTAGTGTGGCCTT--------------------------------
---------------------------CATTCAAGGAGGGTGTGTTGTTCACCACATC
TGCTTCCCACTGCCAAATCAGGCCTCAGAAAGCTTTCTGGAAGTGACGCCAGCTTC
AGGGACAAGGCCCAAGTTTCTAGGGGTCAACACCGTTCCATGGTTCCTG

D)

Deleted sequence in lncMGC-KO5
CATCTGGTAATGTACTACCTGAGGGGGGAGGTGCCGCCTCTCTTTCAGCACCGTGCAACC

E)

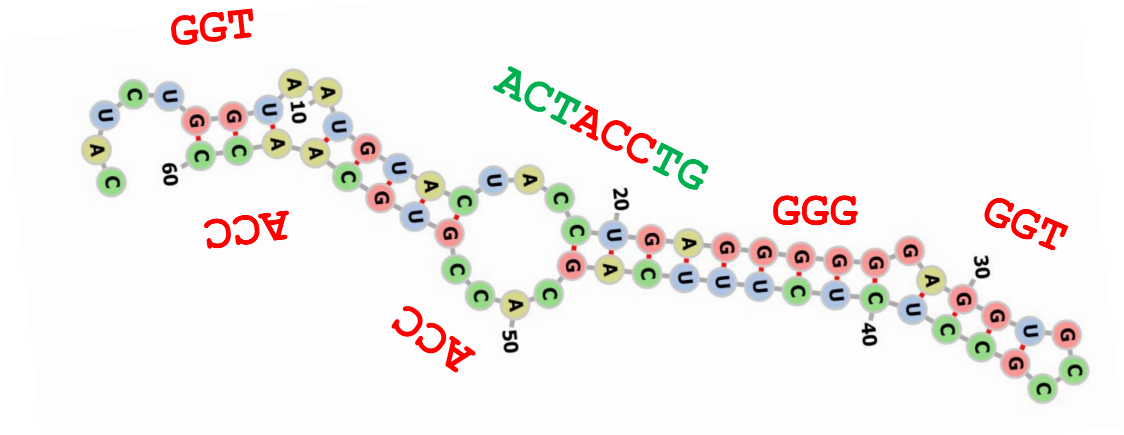

Supplementary Figure 12. ZF and ARID motifs in lncMGC. A, ZF motifs and AT-rich motif in human lncMGC gene. B, ZF motifs and AT-rich motif in mouse lncMGC. C, Sequence of lncMGC in mouse lncMGC-KO5. D, ZF motifs and AT-rich motif in the deleted sequence in mouse lncMGC-KO5. e, Predicted secondary structure of the RNA deleted sequence in lncMGC-KO5. The structure was predicted by the Vienna RNA Websuite (http://rna.tbi.univie.ac.at/). Potential ZF sites (GGT/GGG/ACC/CCC repeats) are in red and AT-rich sequence in green.

A) Overlaps of ATAC peaks and CpG islands

KO > WT | KO< WT | KO = WT

	# up-enriched regions			# Down-enriched regions			# all peaks (Enriched regions)		
	all #	overlapped with CpG islands	250-extended regions overlapped with CpG islands	all	overlapped with CpG islands	250-extended regions overlapped with CpG islands	all #	overlapped with CpG islands	250-extended regions overlapped with CpG islands
**KO.SD vs WT.SD**	1431	207	236	1392	30	34	59660	11455	12519
**KO.TGFb vs WT.TGFb**	446	114	121	518	3	3	37870	9070	9791

B)

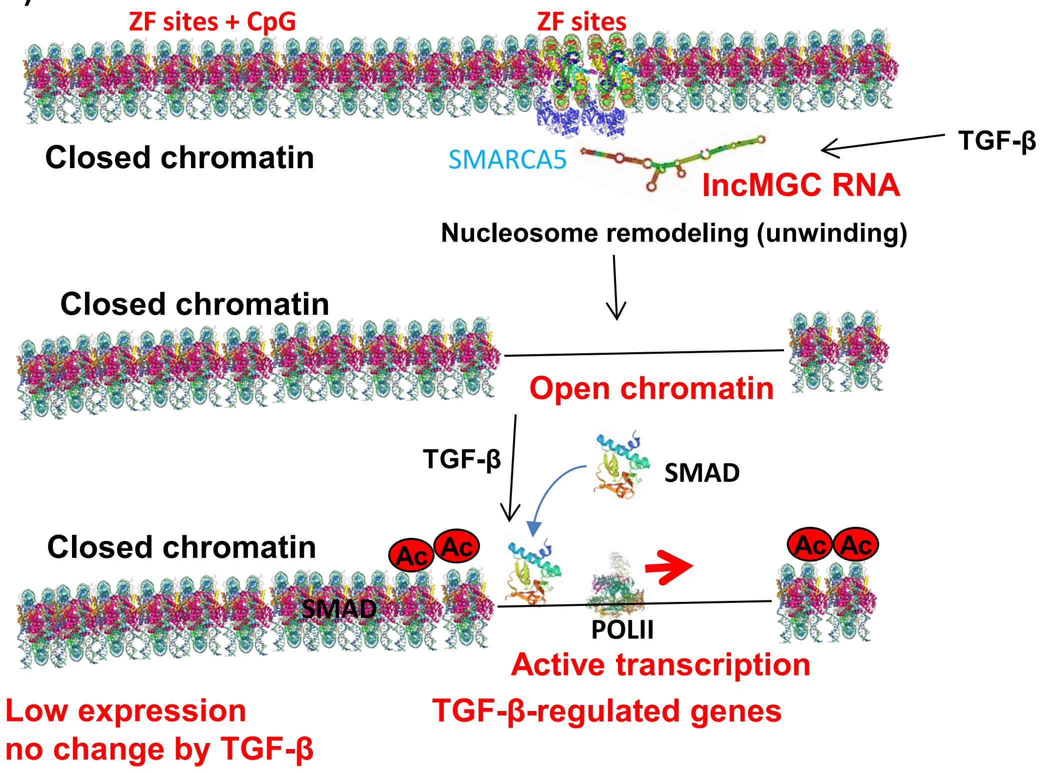

Supplementary Figure 13. Overlaps of ATAC peaks and CpG islands. A, Numbers of overlaps of ATAC-peaks and CpG islands were calculated in up-enriched regions (in KO>WT), down-enriched regions (in KO<WT) and same (in KO=WT) ATAC-peak sites. Overlaps of ATAC-peaks and CpG islands in up-enriched ATAC peak sites in (KO>WT) were significantly greater than those in down-enriched ATAC-peak sites (KO<WT). B, Proposed mechanism of gene regulation by lncMGC mediated by SMARCs and non-CpG-ZF sites. lncMGC-Smrca5 complex may be preferentially recruited to the non-CpG-ZF sites and enhance opening of chromatin and the expression of TGF-β-regulated genes.

Motifs enriched in DEGs (WT_TGF vs WT_SD)

Upregulated genes

Matrix	Accession	Factor name	Classification	P-Value	Yes	No	Yes/No	Matched promoters p-value	Matched promoters in Yes	Matched promoters in No
**V$MZF1_Q5**	**M01733**	**MZF1**	**ZFC2H2**	**1.43E-07**	**0.3094**	**0.2007**	**1.5416**	**1.47E-07**	**0.2702**	**0.1797**
V$EKLF_Q5_01	M07281	KLF1 group	ZFC2H2	2.00E-04	0.0326	0.011	2.964	1.76E-04	0.0326	0.011
V$P53_Q3	M07053	p53 related factors	P53	2.42E-03	0.0078	0.0005	15.6501	2.39E-03	0.0078	0.0005
V$TATA_01	M00252	TBP-related factors	TBP	2.42E-03	0.0078	0.0005	15.6501	2.39E-03	0.0078	0.0005
V$MEF2_03	M00232	Mef-2	MADS	7.39E-03	0.0078	0.001	7.8251	7.31E-03	0.0078	0.001
V$FPM315_01	M01587	FPM315	ZFC2H2	9.61E-03	0.0444	0.0255	1.7389	1.63E-02	0.0392	0.023

Downregulated genes

Matrix	Accession	Factor name	Classification	P-Value	Yes	No	Yes/No	Matched promoters p-value	Matched promoters in Yes	Matched promoters in No
V$CDX2_Q5_02	M02087	CDX group (half-site)	HOX	1.85E-05	0.2617	0.1607	1.6291	3.41E-03	0.1852	0.1311
V$NR3C1_03	M04476	GR-like receptors	ZFC4-NR	1.87E-05	0.0346	0.006	5.7556	2.49E-03	0.0173	0.003
V$MAZ_Q6_01	M02023	MAZ	ZFC2H2	3.65E-05	0.1679	0.0921	1.8232	9.00E-03	0.0765	0.0455
V$IRF1_Q5	M07045	IRF factors	IRF	7.01E-04	0.0123	0.0005	24.6667	3.45E-03	0.0099	0.0005

Supplementary Table 1. Motif analysis (based on differentially expressed genes) enriched in the comparison (WT_TGF vs WT_SD) (FC > 1.5 & p < 5E-3) by GeneXplain (https://genexplain.com/).

Motifs enriched in DEGs (KO_SD versus WT_SD)

Suppl. Table2

Upregulated genes

Matrix	Accession	Factor name	Classification	P-Value	Yes	No	Yes/No	Matched promoters p-value	Matched promoters in Yes	Matched promoters in No
**V$E2F_Q6_01**	**M00920**	**E2F related factors**	**E2F**	**4.32E-06**	**0.0383**	**0.003**	**12.7723**	**2.27E-05**	**0.034**	**0.003**
V$CDX2_Q5_02	M02087	CDX group (half-site)	HOX	1.02E-04	0.2766	0.1604	1.7242	1.22E-02	0.1872	0.1299
V$SIX1_01	M01313	HD-SINE factors	HOX	1.75E-04	0.1745	0.0885	1.9724	6.69E-03	0.1362	0.083
V$NF1A_Q6_01	M03554	NF-1A	SMAD	3.17E-03	0.1447	0.0825	1.7546	2.92E-03	0.1362	0.078
V$RHOX11_01	M01347	Rhox11	HOX	4.28E-03	0.0128	0.0005	25.5447	4.23E-03	0.0128	0.0005
V$RELA_Q6	M03563	NF-kappaB-related factor	REL	4.86E-03	0.0426	0.014	3.041	4.49E-03	0.0426	0.014

Downregulated genes

Matrix	Accession	Factor name	Classification	P-Value	Yes	No	Yes/No	Matched promoters p-value	Matched promoters in Yes	Matched promoters in No
**V$GKLF_Q4**	**M01835**	**KLF4 group**	**ZFC2H2**	**9.14E-08**	**0.9071**	**0.5717**	**1.5866**	**4.03E-02**	**0.4426**	**0.3738**
**V$PBX_Q3**	**M00998**	**Pbx**	**HOX**	**1.27E-06**	**0.0601**	**0.0065**	**9.2522**	**1.45E-01**	**0.0164**	**0.0065**
**V$MZF1_Q5**	**M01733**	**MZF1**	**ZFC2H2**	**7.60E-06**	**0.3607**	**0.1924**	**1.8745**	**3.65E-05**	**0.3005**	**0.1724**
V$CDX2_Q5_02	M02087	CDX group (half-site)	HOX	7.35E-05	0.2951	0.1604	1.8394	1.01E-02	0.1967	0.1299
V$IRF1_Q5	M07045	IRF factors	IRF	2.30E-04	0.0219	0.0005	43.7377	2.17E-03	0.0164	0.0005
V$ZIC1_05	M02939	Zic group	ZFC2H2	1.98E-03	0.0273	0.0035	7.8103	1.90E-03	0.0273	0.0035
V$RREB1_01	M00257	RREB-1	ZFC2H2	2.12E-03	0.2131	0.1244	1.7126	3.25E-02	0.1585	0.1089
V$BBX_03	M02739	Bbx	HMG	4.09E-03	0.0437	0.012	3.6448	6.83E-02	0.0219	0.0075

Supplementary Table 2. Motif analysis (based on differentially expressed genes) enriched in the comparison (KO_SD versus WT_SD) (FC > 1.5 & p < 5E-3) by GeneXplain (https://genexplain.com/).

Motifs enriched in DEGs (KO_TGF vs WT_TGF)

Upregulated genes

Matrix	Accession	Factor name	Classification	P-Value	Yes	No	Yes/No	Matched promoters p-value	Matched promoters in Yes	Matched promoters in No
V$IK_Q5_01	M07260	Ikaros	ZFC2H2	4.89E-04	0.3805	0.2478	1.5358	6.57E-04	0.322	0.2173
V$CP2_Q6	M03868	CP2-related factors	GRAINY	1.96E-03	0.078	0.031	2.5202	3.79E-03	0.0732	0.031
V$GEN_INI_B	M00315	general initiator	GENINI	2.68E-03	0.1902	0.1124	1.6927	2.23E-03	0.1756	0.1039

Downregulated genes

Matrix	Accession	Factor name	Classification	P-Value	Yes	No	Yes/No	Matched promoters p-value	Matched promoters in Yes	Matched promoters in No
**V$GKLF_Q4**	**M01835**	**KLF4 group**	**ZFC2H2**	**7.84E-12**	**0.9286**	**0.538**	**1.7261**	**5.62E-04**	**0.4821**	**0.3671**
**V$RELA_Q6**	**M03563**	**NF-kappaB-related factor**	**REL**	**3.29E-09**	**0.1295**	**0.029**	**4.4688**	**1.03E-07**	**0.1116**	**0.028**
**V$NR3C1_03**	**M04476**	**GR-like receptors**	**ZFC4-NR**	**7.57E-06**	**0.0446**	**0.005**	**8.9375**	**1.63E-03**	**0.0223**	**0.0025**
V$PBX_Q3	M00998	Pbx	HOX	1.95E-05	0.0491	0.0075	6.5542	2.41E-01	0.0134	0.007
V$ZIC1_05	M02939	Zic group	ZFC2H2	3.80E-05	0.0402	0.005	8.0437	3.40E-05	0.0402	0.005
V$CPHX_01	M01478	Cphx	HOX	5.60E-04	0.0268	0.003	8.9375	2.59E-02	0.0134	0.002
V$HOXC13_01	M01317	Hox-13	HOX	2.00E-03	0.2589	0.1668	1.552	5.73E-03	0.2188	0.1494
V$NF1A_Q6_01	M03554	NF-1A	SMAD	2.52E-03	0.1473	0.0819	1.7984	1.29E-02	0.125	0.0774
V$FPM315_01	M01587	FPM315	ZFC2H2	3.40E-03	0.0268	0.005	5.3625	3.27E-03	0.0268	0.005
V$TBX5_Q2	M03856	T-box factors	TBX	3.77E-03	0.0134	0.0005	26.8125	3.73E-03	0.0134	0.0005

Supplementary Table 3. Motif analysis (based on differentially expressed genes) enriched in the comparison (KO_TGF vs WT_TGF) (FC > 1.5 & p < 5E-3) by GeneXplain (https://genexplain.com/).

Motifs enriched in DEGs (KO_TGF vs KO_SD)

Upregulated genes

Matrix	Accession	Factor name	Classification	P-Value	Yes	No	Yes/No	Matched promoters p-value	Matched promoters in Yes	Matched promoters in No
**V$MZF1_Q5**	**M01733**	**MZF1**	**ZFC2H2**	**4.50E-10**	**0.315**	**0.1887**	**1.6694**	**1.28E-08**	**0.2675**	**0.1717**
V$EKLF_Q5_01	M07281	KLF1 group	ZFC2H2	1.02E-04	0.03	0.009	3.33	8.99E-05	0.03	0.009
V$RREB1_01	M00257	RREB-1	ZFC2H2	2.81E-04	0.0412	0.017	2.424	1.17E-03	0.0362	0.016
V$HBP1_03	M02762	Hbp1	HMG	2.89E-03	0.0075	0.0005	14.985	1.98E-01	0.0025	0.0005
V$BBX_03	M02739	Bbx	HMG	3.43E-03	0.025	0.01	2.4975	1.81E-02	0.0138	0.005

Downregulated genes

Matrix	Accession	Factor name	Classification	P-Value	Yes	No	Yes/No	Matched promoters p-value	Matched promoters in Yes	Matched promoters in No
**V$MAZ_Q6_01**	**M02023**	**MAZ**	**ZFC2H2**	**1.22E-09**	**0.2136**	**0.1031**	**2.072**	**1.83E-04**	**0.1105**	**0.0626**
V$RELA_Q6	M03563	NF-kappaB-related factor	REL	2.05E-05	0.0773	0.0325	2.3776	1.49E-04	0.0681	0.031
V$CDX2_Q5_02	M02087	CDX group (half-site)	HOX	8.52E-05	0.2376	0.1577	1.5069	4.97E-04	0.1878	0.1296
V$FPM315_01	M01587	FPM315	ZFC2H2	1.24E-04	0.0442	0.015	2.9436	1.20E-04	0.0387	0.012
V$NR3C1_03	M04476	GR-like receptors	ZFC4-NR	2.83E-04	0.0258	0.006	4.2928	1.00E-02	0.0129	0.003
V$ZIC1_05	M02939	Zic group	ZFC2H2	1.02E-03	0.0203	0.0045	4.4972	9.69E-04	0.0203	0.0045

Supplementary Table 4. Motif analysis (based on differentially expressed genes) enriched in the comparison (KO_TGF vs KO_SD) (FC > 1.5 & p < 5E-3) by GeneXplain (https://genexplain.com/).

The specific negative association of eGFR with DNA methylation of lncMGC loci

eGFR							
probeID	CHR	MAPINFO	Effect estimate	Standard error	P-value	n	
cg18397314	14	1E+08	-3.67E-05	9.11E-06	5.60E-05	33590	
cg01811583	14	1E+08	-4.26E-05	9.35E-06	5.36E-06	33582	
cg01513978	14	1.01E+08	-4.07E-05	8.49E-06	1.58E-06	33585	DLK1
cg02032125	14	1.01E+08	-5.13E-05	1.24E-05	3.57E-05	33588	miR-379
cg11820913	14	1.01E+08	-4.90E-05	1.10E-05	7.97E-06	33586	miR-379
cg17916177	14	1.01E+08	-4.62E-05	1.00E-05	4.10E-06	33588	LncMGC/ MEG9
cg19509303	14	1.01E+08	-3.09E-05	7.42E-06	3.09E-05	33520	
cg21230021	14	1.02E+08	-3.86E-05	8.94E-06	1.54E-05	33569	DIO3
cg20547131	14	1.02E+08	-4.26E-05	9.50E-06	7.56E-06	33586	DIO3
cg11789371	14	1.03E+08	-3.61E-05	7.90E-06	4.79E-06	33591	
cg13496568	14	1.03E+08	3.27E-05	8.29E-06	7.94E-05	33588	
cg04987734	14	1.03E+08	9.16E-05	1.65E-05	2.71E-08	32070	MARK3
UACR							
probeID	CHR	MAPINFO	Effect estimate	Standard error	P-value	n	
cg01513978	14	1.01E+08	0.000174	0.000164	0.288695	14498	DLK1
cg02032125	14	1.01E+08	-9.45E-05	0.000286	0.740648	14502	miR-379
cg11820913	14	1.01E+08	-0.00024	0.000235	0.313998	14498	miR-379
cg17916177	14	1.01E+08	-0.00012	0.000215	0.567875	14503	lncMGC/ MEG9
cg19509303	14	1.01E+08	-0.00027	0.000138	0.048889	14439	
cg21230021	14	1.02E+08	-0.00014	0.000177	0.43703	14486	DIO3
cg20547131	14	1.02E+08	-8.73E-05	0.000207	0.67258	14502	DIO3
CKD							
probeID	CHR	MAPINFO	Effect estimate	Standard error	P-value	n	
cg01513978	14	1.01E+08	0.000543	0.000562	0.334198	25599	DLK1
cg02032125	14	1.01E+08	0.002604	0.000824	0.001572	25598	miR-379
cg11820913	14	1.01E+08	0.002063	0.000679	0.002386	25595	miR-379
cg17916177	14	1.01E+08	0.001486	0.000614	0.015488	25598	lncMGC/ MEG9
cg19509303	14	1.01E+08	0.000921	0.000499	0.064904	25568	
cg21230021	14	1.02E+08	0.0014	0.000598	0.019164	25587	DIO3
cg20547131	14	1.02E+08	0.001542	0.000609	0.011294	25595	DIO3

Supplementary Table 5. Re-analysis of Meta data (N=33605) on DNA methylation associated with kidney function and damage[6]. A negative association of eGFR (estimated glomerular filtration rate) with lncMGC loci was detected but no association with UACR (Urinary albumin to creatinine ratio) and CKD (chronic kidney disease) was noted. . Meta-analyses identify DNA methylation associated with kidney function and damage. (Schlosser et al., *Nature communications* **12**, 7174, 2021).

Human		
hlncMGC	Forward	GCCTGCTTCCAATGCCAAATC
	Reverse	CTTCAGGAACCACGGAATGGT
NRON	Forward	ACGTTCCTTAATGTACGCCTTTGC
	Reverse	TTGGCCGTGTCCTGAGTCCTT
lncTCF7	Forward	AGGAGTCCTTGGACCTGAGC
	Reverse	AGTGGCTGGCATATAACCAACA
PURPL	Forward	CGTGTGAAAAGAACCCAGGTA
	Reverse	CGCCTGGTAAAACAACCAGT
MALAT1	Forward	GACGGAGGTTGAGATGAAGC
	Reverse	ATTCGGGGCTCTGTAGTCCT
7SKRNA	Forward	GGATGTGAGGGCGATCTG
	Reverse	GGAGCGGTGAGGGAGGAAG
TSC22	Forward	AAGCCATTTGATGTATGCGG
	Reverse	GATTGTTCTCCTGCTCCAGCT
hCypA (PPIA)	Forward	CCCACCGTGTTCTTCGACATT
	Reverse	GGACCCGTATGCTTTAGGATGA
mouse		
lncMGC INR	Forward	ATTTTTCTGAGTTAGTGTGGCCTTCATCTG
	Reverse	TCAGGAACCATGGAACGGTGTTGACCCCTAG
mCol4a3	Forward	CAAAGGCATCAGGGGAATAACT
	Reverse	ATCCGTTGCATCCTGGTAAAC
mCol4a4	Forward	ATGAGGTGCTTTTTCAGATGGAC
	Reverse	GGGGCCGCCATACTTCTTG
mUcp2	Forward	ATGGTTGGTTTCAAGGCCACA
	Reverse	CGGTATCCAGAGGGAAAGTGAT
mNkd2	Forward	GAGCGGAAGAAACGGACCG
	Reverse	CCTTAGGGTCTCCATTGAGCA
mNlrx1	Forward	TAGGGCCTTTATCCGTTACCA
	Reverse	TAAACCACTCGGTGAGGTTCC
mSmarca5	Forward	GACACCGAGATGGAGGAAGTA
	Reverse	CGAACAGCTCTGTCTGCTTTA
Nox4	Forward	TGTTGGGCCTAGGATTGTGTT
	Reverse	AGGGACCTTCTGTGATCCTCG
Meg3	Forward	TCCTCACCTCCAATTTCCCCT
	Reverse	GAGCGAGAGCCGTTCGATG
Rian	Forward	TGTCACGGTCAGCTCTGTTC
	Reverse	ACCAAGGTGTACGCAACGAT
Dlk1	Forward	AGCACCTATGGGGCTGAATG
	Reverse	CACTTGTCACAGAGGGGACC
Mirg	Forward	CCTTCCTGGATCTCTCGCTT
	Reverse	GTGGGAGTTGAAACATGGGT
Pai1	Forward	GCAGCTCTCTGTAGCACAAGCA
	Reverse	CGGCCTCTGTTGGATTGTGCCG
CypA (Ppia)	Forward	ATGGTCAACCCCACCGTGT
	Reverse	TTCTTGCTGTCTTTGGAACTTTGTC
ChIP primers		
Smad site	Forward	GAGAATCTACAGAGACTGAGAATCTGCACATG
	Reverse	GGTCTGAAACATCTCCATCCAGTCTGG
Chop site	Forward	GAGCTCTTGCTCTTTGCACCTGCG
	Reverse	AAGCAGGTGGAACCAGAAGTAAGCC
lncMGC INR	Forward	ATTTTTCTGAGTTAGTGTGGCCTTCATCTG
	Reverse	TCAGGAACCATGGAACGGTGTTGACCCCTAG

Supplementary Table 6. PCR primers. used for qPCR in this study
